# Effects of cottonseed meal protein hydrolysate on intestinal microbiota of yellow-feather broilers

**DOI:** 10.3389/fmicb.2024.1434252

**Published:** 2024-09-18

**Authors:** Xiaoyang Zhang, Hailiang Wang, Yujie Niu, Cheng Chen, Wenju Zhang

**Affiliations:** College of Animal Science and Technology, Shihezi University, Shihezi, China

**Keywords:** cottonseed meal protein hydrolysate, yellow-feather broilers, intestinal microbiota, microbial diversity and richness, microbial network

## Abstract

We evaluated the effects of cottonseed meal protein hydrolysate (CPH) on the intestinal microbiota of yellow-feather broilers. We randomly divided 240 chicks into four groups with six replicates: basal diet with 0% (CON), 1% (LCPH), 3% (MCPH), or 5% (HCPH) CPH. The test lasted 63 days and included days 1–21, 22–42, and 43–63 phases. The ACE, Chao1, and Shannon indices in the MCPH and HCPH groups of 42-day-old broilers were higher than those in the CON group (*p* < 0.05), indicating that the cecum microbial diversity and richness were higher in these groups. *Firmicutes* and *Bacteroidetes* were the dominant phyla; however, the main genera varied during the different periods. The abundance of *Lactobacillus* in CPH treatment groups of 21-day-old broilers was high (*p* < 0.05); in the 42-day-old broilers, the abundances of *Barnesiella*, *Clostridia_vadinBB60_group*, and *Parasutterella* in the LCPH group, *Desulfovibrio*, *Lactobacillus*, *Clostridia_vadinBB60_group*, and *Butyricicoccus* in the MCPH group, and *Megamonas* and *Streptococcus* in the HCPH group increased; in the 63-day-old broilers, the abundance of *Clostridia_UCG-014* and *Synergistes* in the LCPH and HCPH group, respectively, increased (*p* < 0.05), and that of *Alistipes* in the LCPH and MCPH groups decreased (*p* < 0.05). And changes in the abundance of probiotics were beneficial to improve the intestinal morphology and growth performance. In addition, the LCPH treatment increased the complexity of the microbial network, while the MCPH treatment had the same effect in 42-day-old broilers. Thus, CPH increased the relative abundance of beneficial intestinal microbiota and enhanced the richness and diversity of the bacterial microbiota in broilers aged <42 days; this effect was weakened after 42 days.

## 1 Introduction

Antibiotics are widely used in poultry production; however, their continued use can lead to the emergence of antibiotic-resistant pathogens, which in turn can adversely affect human and animal health (Zhang et al., 2022). Therefore, the development of substitutes for antibiotics for animal husbandry has attracted considerable attention, with protein hydrolysates (PH) mooted as possible replacements for antibiotics (Zhang et al., 2024). PH, which can be produced by enzymatic hydrolysis, contribute largely to the health benefits of food (Tang et al., 2023). Owing to its efficiency and safety, enzymatic hydrolysis is currently one of the most promising methods for preparing peptides. As shown previously, PH may contain prebiotic peptides such as tripeptides (Glu-Leu-Met) (Arihara, 2006; Brody, 2000; Safari et al., 2012). Cottonseed meal protein hydrolysate (CPH) is a product of the enzymolysis of cottonseed meal protein, which possesses antioxidant, antibacterial, angiotensin-I-converting enzyme inhibitory activities and benefificial pharmacological properties (de Oliveira Filho et al., 2021; Gao et al., 2010; Mahdavi-Yekta et al., 2023; Ozturk-Kerimoglu et al., 2023; Sharma and Thakur, 2024; Song et al., 2020; Wang et al., 2021) and exerts a positive effect on animals (Alizadeh-Ghamsari et al., 2023; Cheng et al., 2019; Gisbert et al., 2021; Gui et al., 2010; Hartnoll, 2001; Tolba et al., 2023); thus, it is expected to be one of substitutes for antibiotics.

The intestinal microbiota plays an important role in animals, and the poultry intestinal microbiota responds to environmental changes (Ma et al., 2021; Singh et al., 2022; Stanley et al., 2013, 2014). The human microbiota can respond rapidly to changes in diet, which is the most influential variable (Candela et al., 2012; Turnbaugh et al., 2009). The effect of the intestinal microbiota on weight gain and productivity has been well documented in both humans and poultry (Abudabos et al., 2017; Ridaura et al., 2013; van Kuijk et al., 2021). The cecum, an important part of the gastrointestinal tract that is the site of most fermentations, significantly affects intestinal health and animal nutrition (Zhang et al., 2022).

Although CPH has been studied in the feed of agricultural animals, to date, its effects on the intestinal microbial populations in chickens have not been evaluated. This study was designed to evaluate the effects of graded levels of CPH on the intestinal microbial populations of yellow-feather broilers and provide a theoretical reference for the development of biological protein feeds.

## 2 Materials and methods

### 2.1 Ethics statement

Ethical requirements and recommendations for animal care for the experiment were approved by the Biology Ethics Committee of Shihezi university (Xinjiang, China) (no. A2021-47).

### 2.2 Preparation of CPH

Cottonseed meal protein (CP; protein purity, 91.03%) was isolated from cottonseed meal and hydrolyzed using 1:1 ratio of neutral protease (CAS:9068-59-1; Beijing Solarbio Science and Technology Co. Ltd., China) and papain (CAS:9001-73-4; Beijing Biotoppted Science and Technology Co. Ltd., China) (each enzyme/substrate ratio was 6,000 IU g^−1^) at 49.65°C and pH 6.99 for 2.83 h. CPH was heated at 95°C for 10 min to stop the reaction and was freeze-dried.

### 2.3 Diets and experimental design

The experimental diets used in this study are shown in Table 1. Four experimental diets supplemented with different concentrations (0, 1, 3, and 5%) of CPH were formulated for each trial period. Low CPH (LCPH), Medium CPH (MCPH) and High CPH (HCPH) group were supplemented with 1, 3 and 5% CPH, respectively. In total of 240 1-day-old healthy male yellow-feather chickens (initial weight 40.75 ± 1.27 g) were purchased from a commercial breeding base (Shihezi, China) and randomly distributed into four groups with six replicates of 10 birds. The test lasted 63 days and included three phases: days 1–21, 22–42, and 43–63.

**Table 1:** Composition of the diets and nutrient levels.

Item	1–21				22–42				43–63			
	CON	LCPH	MCPH	HCPH	CON	LCPH	MCPH	HCPH	CON	LCPH	MCPH	HCPH
Ingredients												
Corn	58.17	58.11	58.03	57.94	65.89	65.83	65.76	65.65	70.00	69.97	69.89	69.79
Soybean meal	30.28	30.33	30.38	30.45	21.98	22.02	22.07	22.15	16.74	16.76	16.82	16.89
Cottonseed meal protein	5.00	4.00	2.00	0.00	5.00	4.00	2.00	0.00	5.00	4.00	2.00	0.00
CPH	0.00	1.00	3.00	5.00	0.00	1.00	3.00	5.00	0.00	1.00	3.00	5.00
Soya-bean oil	1.55	1.56	1.59	1.61	2.13	2.15	2.17	2.20	3.26	3.27	3.29	3.32
Premix	5.00	5.00	5.00	5.00	5.00	5.00	5.00	5.00	5.00	5.00	5.00	5.00
Total	100.00	100.00	100.00	100.00	100.00	100.00	100.00	100.00	100.00	100.00	100.00	100.00
**Nutrient levels**												
Chemical compositions												
Metabolizable energy, MJ/kg	12.13	12.13	12.13	12.13	12.55	12.55	12.55	12.55	12.98	12.98	12.98	12.98
Crude protein	23.00	23.00	23.00	23.00	20.00	20.00	20.00	20.00	18.04	18.04	18.04	18.04
Calcium	1.00	1.00	1.00	1.00	0.90	0.90	0.90	0.90	0.80	0.80	0.80	0.80
Available phosphate	0.45	0.45	0.45	0.45	0.35	0.35	0.35	0.35	0.30	0.30	0.30	0.30
Lysine	1.10	1.10	1.10	1.11	1.00	1.00	1.00	1.00	0.85	0.85	0.85	0.85

Premix provided the following per kg of diets: 1) 1-21 d, Ca 8.80 g; P 3.00 g; NaCl 2 g; Lys 0.05 g; Met 1.7 g; VA 1000 IU; VD3 200 IU; VE 8 IU; VK3 2.2 mg; VB1 1.5 mg; VB2 3.0 mg; VB3 8.0 mg; VB4 300 mg; VB5 25 mg; VH 0.10 mg; VB11 0.20 mg; VB12 0.02 mg; VC 50 mg; Mn 65 mg; Zn 45 mg; Cu 8 mg; Se 0.20 mg; I 0.40 mg; Fe 80 mg. 2) 22-42 d, Ca 8.00 g; P 2.05 g; NaCl 1.5 g; Lys 1.10 g; Met 0.9 g; VA 1000 IU; VD3 200 IU; VE 8 IU; VK3 2.0 mg; VB1 1.5 mg; VB2 3.0 mg; VB3 8.0 mg; VB4 200 mg; VB5 20 mg; VH 0.10 mg; VB11 0.20 mg; VB12 0.02 mg; VC 50 mg; Mn 65 mg; Zn 45 mg; Cu 8 mg; Se 0.20 mg; I 0.40 mg; Fe 80 mg. 3) 43-63 d, Ca 7.15 g; P 1.60 g; NaCl 1.2 g; Lys 0.75 g; Met 0.5 g; VA 1000 IU; VD3 200 IU; VE 8 IU; VK3 0.4 mg; VB1 1.5 mg; VB2 2.5 mg; VB3 8.0 mg; VB4 100 mg; VB5 15 mg; VH 0.08 mg; VB11 0.18 mg; VB12 0.02 mg; VC 50 mg; Mn 65 mg; Zn 45 mg; Cu 8 mg; Se 0.20 mg; I 0.40 mg; Fe 80 mg. Nutrient levels were all calculated values.

### 2.4 Gut microbiota study

#### 2.4.1 DNA extraction and polymerase chain reaction (PCR)

Cecal samples were collected immediately after the broilers were euthanized. Total bacterial DNA was extracted from the cecal samples using the E.Z.N.A.^®^ soil DNA kit (Omega Bio-Tek, Norcross). The quantity and quality of the extracted DNA were determined by electrophoresing on a 1.0% agarose gel and using a NanoDrop 2000 UV–visible spectrophotometer, respectively (Thermo Scientific, Wilmington, NC, United States). The V3-V4 region of the 16S rRNA was amplified using PCR with forward (ACTCCTACGGGAGGCAGCAG) and reverse primers (GGACTACHVGGGTWTCTAAT). The PCR mixture included 5× FastPfu buffer (4 μL), dNTPs (2 μL), primers (0.8 μL × 2), FastPfu polymerase (0.4 μL), bovine serum albumin (0.2 μL), DNA template (10 ng), and ddH2O (to make up to a final volume of 20 μL). The amplification conditions were as follows: initial denaturation at 95°C for 3 min; 27 cycles of denaturation at 95°C for 30 s, 55°C for 30 s, and 72°C for 45 s; followed by holding at 72°C for 10 min. The resulting PCR products were analyzed using electrophoresis on a 2% agarose gel.

#### 2.4.2 High-throughput sequencing

The purified amplicons were pooled in equimolar amounts, and double-end sequencing was performed using the Illumina MiSeq PE300 platform (Illumina, San Diego, CA, United States) according to the standard protocols of Majorbio Bio-Pharm Technology Co., Ltd. (Shanghai, China). The sequences were submitted to NCBI under the accession number PRJNA1031419.

#### 2.4.3 Bioinformatics analysis

Operational taxonomic units (OTUs) were obtained based on a 97% similarity cut-off using UPARSE (version 11) after mass filtering and removal of chimeric sequences. The taxonomy of each 16S rRNA gene sequence was analyzed using the Ribosomal Database Project Classifier algorithm (version 11.5) against the Silva 16S rRNA database (version 138).

### 2.5 Statistical analysis

Bioinformatics analysis of the gut microbiota was performed using the Majorbio Cloud platform. Based on the OTU information, rarefaction curves and alpha diversity indices were calculated using Mothur (version 1.30.2). Similarity was determined using principal coordinate analysis (PCoA) based on Bray–Curtis dissimilarity in the Vegan package of the R (V3.3.1) software. Non-metric multidimensional scaling (NMDS) analysis was conducted using the Vegan package in the R (V3.3.1) software. Partial least squares discriminant analysis (PLS-DA) was performed using the mixOmics package in the R (V3.3.1) software. The correlation network of cecal microorganisms was constructed using Networkx software (version 1.11).

## 3 Results and analysis

### 3.1 Sequencing results of the cecal microflora

**Figure 1:**
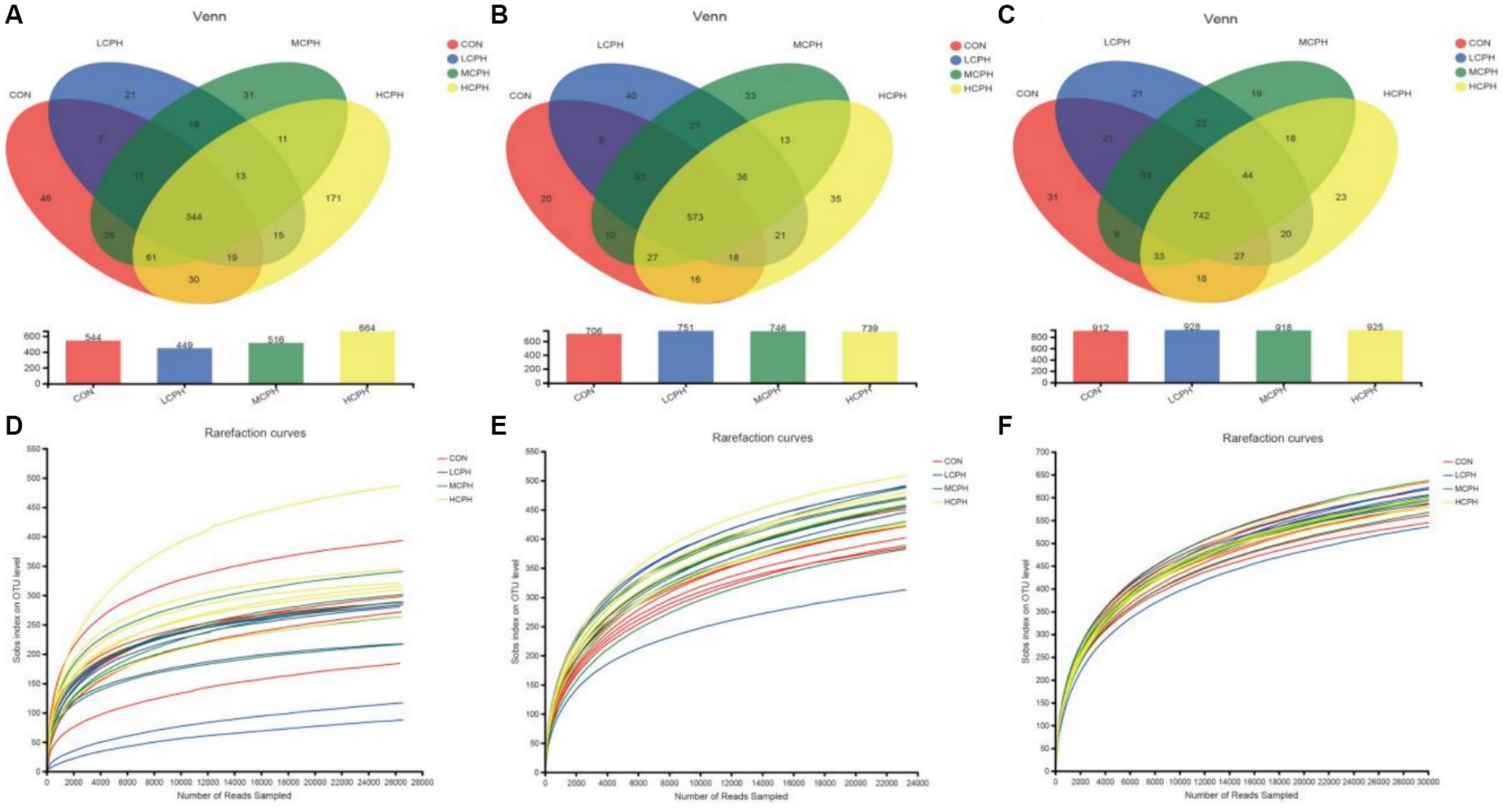
Venn diagrams and dilution curve of dietary treatments at the OTUs level. **(A–C)** Venn diagrams in 21-day-old, 42-day-old, and 63-day-old yellow-feather broilers, respectively. **(D–F)** Dilution curve in 21-day-old, 42-day-old, and 63-day-old yellow-feather broilers, respectively. CON, basal diet; LCPH, diet with 1% CPH; MCPH, diet with 3% CPH; HCPH, diet with 5% CPH.

In this study, 3,588,955 high-quality reads were acquired from 72 cecal chyme samples, with an average of 49,847 sequences per sample. There were 1,079,687, 1,206,267, and 1,303,001 high-quality reads with averages of 44,987, 50,261, and 54,292 sequences per sample in 21-, 42-, and 63-day-old yellow-feather broilers, respectively. As shown in Figure 1, after quality and chimera checking, 344 shared OTUs were determined (with 97% similarity) in 21-day-old broilers, and 46, 21, 31, and 171 characteristic OTUs were found for in the CON, LCPH, MCPH, and HCPH groups, respectively; 573 shared OTUs were determined (with 97% similarity) in 42-day-old broilers, and 20, 40, 33, and 35 characteristic OTUs were found for in the CON, LCPH, MCPH, and HCPH groups, respectively; and 742 shared OTUs were determined (with 97% similarity) in 63-day-old broilers, and 31, 21, 19, and 23 characteristic OTUs were found in the CON, LCPH, MCPH, and HCPH groups, respectively. The dilution curve of each sample reached a plateau stage, indicating that the sequencing depth was sufficient to reflect the vast majority of microbial diversity information in the samples.

### 3.2 Diversity analysis of cecal microbiota

#### 3.2.1 Effect of CPH on cecal microbial alpha diversity

**Figure 2:**
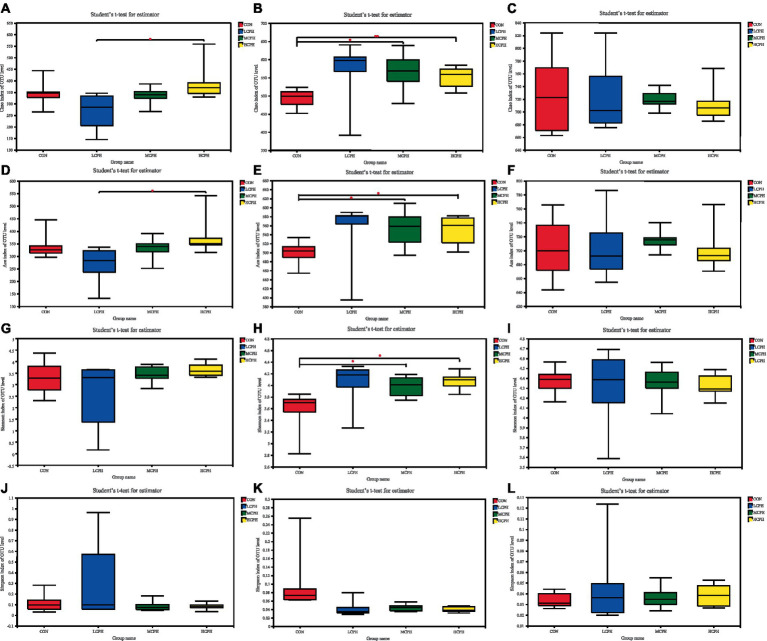
The bacterial *α*-diversity of the cecum in broilers dietary with CPH supplementation. **(A–C)** Chao index of cecal microbiota in 21-day-old, 42-day-old, and 63-day-old yellow-feather broilers, respectively. **(D–F)** Ace index of cecal microbiota in 21-day-old, 42-day-old, and 63-day-old yellow-feather broilers, respectively. **(G–I)** Shannon index of cecal microbiota in 21-day-old, 42-day-old, and 63-day-old yellow-feather broilers, respectively. **(J–L)** Simpson index of the OTUs community of cecal microbiota in 21-day-old, 42-day-old, and 63-day-old yellow-feather broilers, respectively. CON, basal diet; LCPH, diet with 1% CPH; MCPH, diet with 3% CPH; HCPH, diet with 5% CPH.

The effects of CPH on cecal microbial alpha diversity are shown in Figure 2. The coverage rate of each group was >99%, indicating that the probability of sequence detection in the sample was very high, whereas that of not being detected was very low. The sequencing results represented the actual conditions of the microorganisms in the sample. Alpha diversity represents the abundance and diversity of species in each sample. No significant differences were observed in the ACE, Chao1, Simpson, and Shannon indices of the LCPH, MCPH, and HCPH groups compared with that of the CON group, while the ACE and Chao1 indices of the HCPH group were significantly higher than those of the LCPH group in 21-day-old broilers. The ACE, Chao1 and Shannon indices of the MCPH and HCPH groups were significantly higher than those of the CON group, whereas the Simpson index did not differ significantly among the four treatments, indicating that cecum microbial diversity and richness were higher in the MCPH and HCPH groups of the 42-day-old broilers. Alpha diversity did not differ significantly among the four treatments in 63-day-old broilers.

#### 3.2.2 Effect of CPH on cecal microbial beta diversity

**Figure 3:**
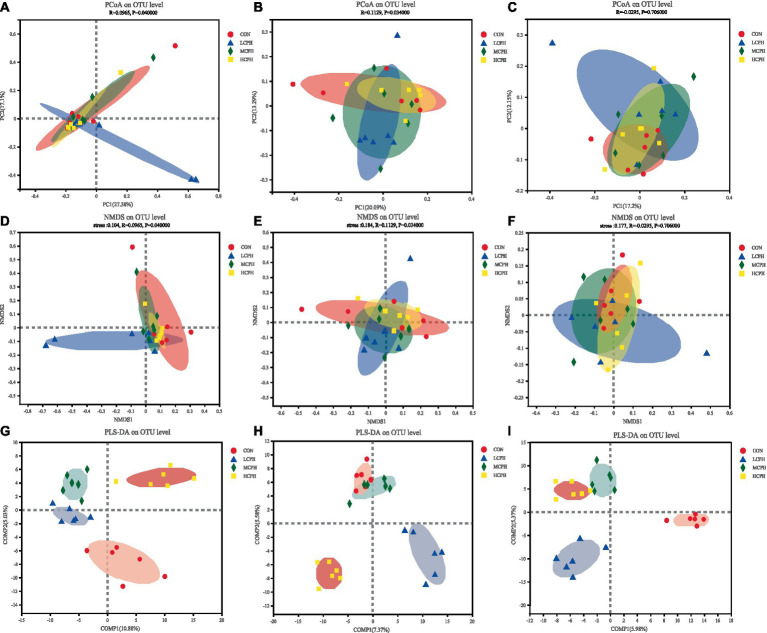
The bacterial *β*-diversity of the cecum in broilers dietary with CPH supplementation. **(A–C)** Principal coordinates analysis (PCoA) of cecal microbiota (based on the Bray distance) in 21-day-old, 42-day-old, and 63-day-old yellow-feather broilers, respectively. **(D–F)** NMDS analysis of cecal microbiota in 21-day-old, 42-day-old, and 63-day-old yellow-feather broilers, respectively. **(G–I)** PLS-DA analysis of cecal microbiota in 21-day-old, 42-day-old, and 63-day-old yellow-feather broilers, respectively. CON, basal diet; LCPH, diet with 1% CPH; MCPH, diet with 3% CPH; HCPH, diet with 5% CPH.

Beta diversity analysis revealed the natural distribution of the samples, reflecting their similarity. The effects of CPH on cecal microbial beta diversity are shown in Figure 3. PCoA with Bray–Curtis distance was used to analyze the cecal microflora structure in the treatment groups. To further analyze the overall structural changes in the gut microbiota among the different groups, an NMDS diagram based on the Bray–Curtis distance was constructed.

In 21-day-old broilers, PCoA indicated that the LCPH group tended to be distinct from the CON group, although this was not the case for the other treatment groups. The greater the distance between the sample points in the NMDS diagram, the lower similarity of the samples. The bacterial members showed higher differences in the LCPH group than in the CON group. Points in the MCPH and HCPH groups were more concentrated than those in the CON group. In addition, PLS-DA indicated that the treatment groups could be clearly distinguished, and that there was no sample crossover between the groups.

In 42-day-old broilers, PCoA indicated that the LCPH and MCPH groups tended to be distinct from the CON group, and that the points in the HCPH group were more concentrated than those in the CON group, suggesting that CPH changed the intestinal flora in the characteristic direction. The sample points in the LCPH, MCPH, and HCPH groups were more concentrated than those in the CON group in the NMDS diagram, indicating that the samples in the LCPH, MCPH, and HCPH groups were highly similar. As shown in the PLS-DA plot, the LCPH and HCPH groups were clearly distinguishable from the CON group, with no sample crossover.

In 63-day-old broilers, PCoA indicated that the CPH-supplemented groups differed from the CON group. The NMDS diagram showed that the samples distribution within the LCPH, MCPH, and HCPH groups were more dispersed than that in the CON group. PLS-DA indicated that the treatment groups could be clearly distinguished and tha sample crossover between the groups was absent.

### 3.3 Analysis of the composition of cecal microbiota

**Figure 4:**
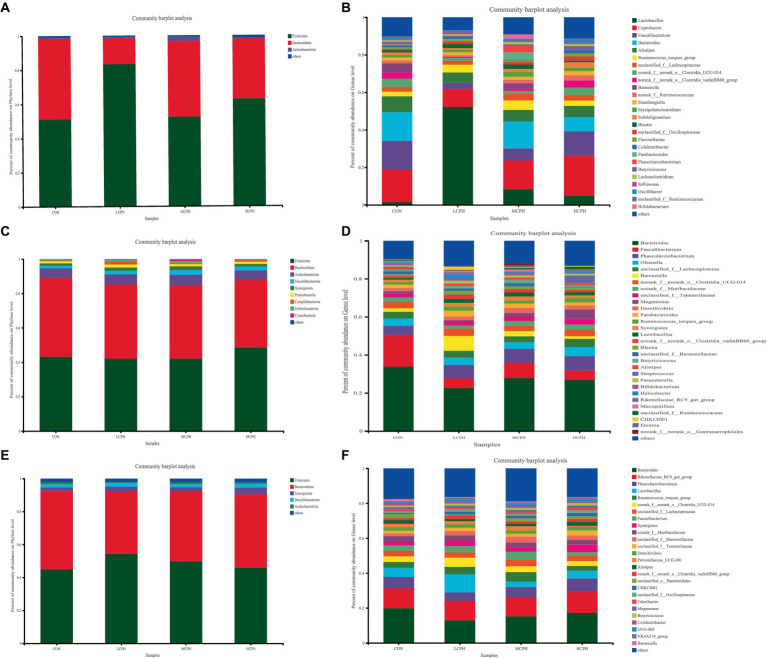
Relative abundance of the broilers’ caecal microbiota in level phylum and genus. **(A,B)** Relative abundance of the broilers’ caecal microbiota in level phylum and genus in 21-day-old yellow-feather broilers. **(C,D)** Relative abundance of the broilers’ caecal microbiota in level phylum and genus in 42-day-old yellow-feather broilers. **(E,F)** Relative abundance of the broilers’ caecal microbiota in level phylum and genus in 63-day-old yellow-feather broilers. Each mean represents six samples. CON, basal diet; LCPH, diet with 1% CPH; MCPH, diet with 3% CPH; HCPH, diet with 5% CPH.

The results of the analysis of the phylum-level composition in broiler chickens is shown in Figure 4 and Supplementary Table S1. *Firmicutes*, *Bacteroidetes*, and *Actinobacteriota* were the top three phyla in 21-day-old broilers, and *Firmicutes* and *Bacteroidetes* accounted for 62.46 and 35.59% of the population, respectively. In addition, based on the analysis of the microflora composition, 246 genus-level microbial taxa were obtained, including 100 genera in all samples and 20 taxa with high abundance: *Lactobacillus*, *Coprobacter*, *Faecalibacterium*, *Bacteroides*, *Alistipes*, *Ruminococcus_torques_group*, *unclassified_f__Lachnospiraceae*, *Clostridia_UCG-014*, *Clostridia_vadinBB60_group*, *Barnesiella*, *Ruminococcaceae*, *Eisenbergiella*, *Erysipelatoclostridium*, *Subdoligranulum*, *Blautia*, *unclassified_f__Oscillospiraceae*, *Flavonifractor*, *Colidextribacter*, *Phascolarctobacterium*, and *Butyricicoccus* (Supplementary Table S2).

In 42-day-old broilers, the top nine phyla identified were Firmicutes, Bacteroidetes, Actinobacteriota, Desulfobacteriota, Synergistota, Proteobacteria, Campylobacterota, Deferribacterota, and Cyanobacteria. Among these, Firmicutes, Bacteroidetes, and Actinobacteriota were the dominant phyla, accounting for 44.03, 42.78, and 5.73% of the population, respectively. In addition, based on the analysis of the microflora composition, 170 genera were identified, including 132 genera in all samples and 22 taxa with high abundance: *Bacteroides*, *Faecalibacterium*, *Phascolarctobacterium*, *Olsenella*, *unclassified_f__Lachnospiraceae*, *Barnesiella*, *Clostridia_UCG-014*, *Muribaculaceae*, *unclassified_f__Tannerellaceae*, *Megamonas*, *Desulfovibrio*, *Parabacteroides*, *Ruminococcus_torques_group*, *Synergistes*, *Lactobacillus*, *Clostridia_vadinBB60_group*, *Blautia, unclassified_f__Barnesiellaceae*, *Butyricicoccus*, *Alistipes*, *Streptococcus*, and *Parasutterella* (Supplementary Table S3).

In 63-day-old broilers, the top nine phyla identified were *Firmicutes*, *Bacteroidetes*, *Synergistota*, *Desulfobacteriota*, and *Actinobacteria*. And *Firmicutes* and *Bacteroidetes* accounted for 48.84 and 43.03% of the population, respectively. In addition, based on the analysis of the microflora composition, 204 genus-level microbial taxa were obtained, including 160 genera in all samples and 24 taxa with high abundance: *Bacteroides*, *Rikenellaceae_RC9_gut_group*, *Phascolarctobacterium*, *Lactobacillus*, *Ruminococcus_torques_group*, *Clostridia_UCG-014*, *unclassified_f__Lachnospiraceae*, *Faecalibacterium*, *Synergistes*, *Muribaculaceae*, *unclassified_f__Barnesiella*, *unclassified_f__Tannerellaceae*, *Desulfovibrio*, *Prevotellaceae_UCG-001*, *Alistipes*, *Clostridia_vadinBB60_group*, *unclassified_o__Bacteroidales*, *CHKCI001*, *unclassified_f__Oscillospiraceae*, *Odoribacter*, *Megamonas*, *Butyricicoccus*, *Colidextribacter*, and *UCG-005* (Supplementary Table S4).

**Figure 5:**
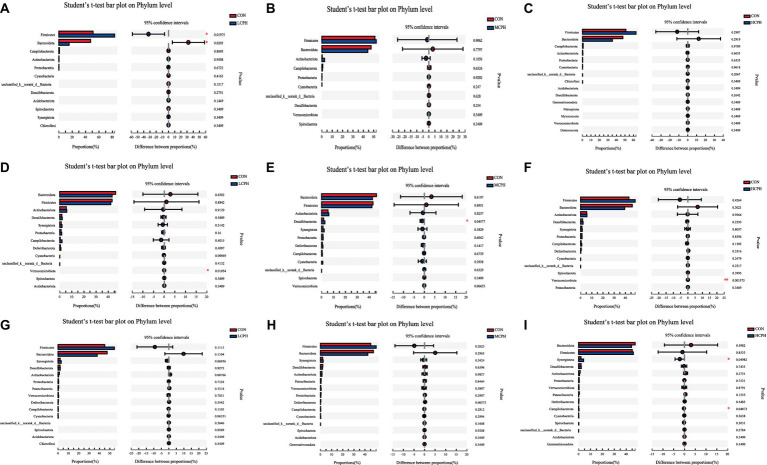
One-way ANOVA analysis of bacterial composition at the phylum level. **(A–C)** One-way ANOVA analysis of bacterial composition at the phylum level in 21-day-old yellow-feather broilers. **(D–F)** One-way ANOVA analysis of bacterial composition at the phylum level in 42-day-old yellow-feather broiler. **(G–I)** One-way ANOVA analysis of bacterial composition at the phylum level in 63-day-old yellow-feather broiler. The asterisk (*) level presented the degree of significant difference, *p < 0.05, **p < 0.01, and ***p < 0.001. Each mean represents six samples. CON, basal diet; LCPH, diet with 1% CPH; MCPH, diet with 3% CPH; HCPH, diet with 5% CPH.

**Figure 6:**
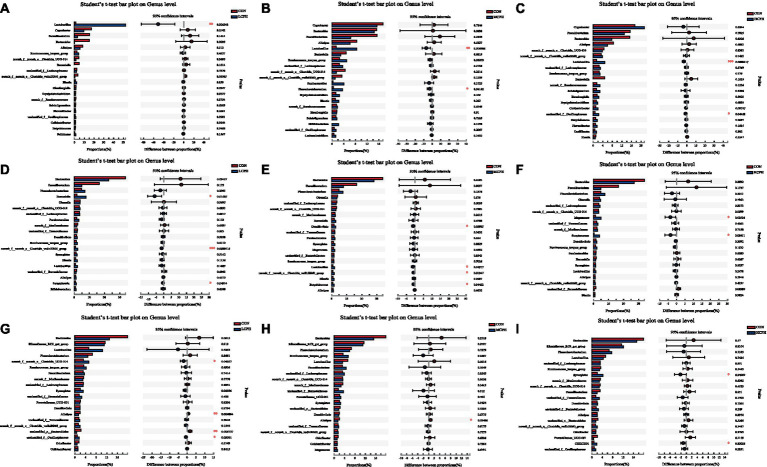
One-way ANOVA analysis of bacterial composition at the genus level. **(A–C)** One-way ANOVA analysis of bacterial composition at the genus level in 21-day-old yellow-feather broilers. **(D–F)** One-way ANOVA analysis of bacterial composition at the genus level in 42-day-old yellow-feather broilers. **(G–I)** One-way ANOVA analysis of bacterial composition at the genus levelin 63-day-old yellow-feather broilers. The asterisk (*) level presented the degree of significant difference, *p < 0.05, **p < 0.01, and ***p < 0.001. Each mean represents six samples. CON, basal diet; LCPH, diet with 1% CPH; MCPH, diet with 3% CPH; HCPH, diet with 5% CPH.

In 21-day-old broilers, *Firmicutes* were more abundant in the LCPH group (*p* < 0.05), whereas *Bacteroidetes* were more abundant in the CON group (*p* < 0.10) (Figure 5). At the genus level, the LCPH group showed higher relative proportion of *Lactobacillus* (*p* < 0.05) and a trend towards decreased relative abundance of *Clostridia_vadinBB60_group* (*p* < 0.10) than that in the CON group. *Lactobacillus* and *Phascolarctobacterium* were more abundant in the MCPH group than in the CON group (*p* < 0.05), and the abundance of *Ruminococcus_torques_group* tended to decrease (*p* < 0.10). The HCPH group showed higher relative proportions of *Lactobacillus* (*p* < 0.05) and a trend for increased abundance of *Colidextribacter* compared to that in CON group (*p* < 0.10) (Figure 6).

In 42-day-old broilers, the abundance of *Verrucomicrobiota* in the LCPH and HCPH groups was higher than that in the CON group (*p* < 0.05), whereas the abundance of *Verrucomicrobiota* in the MCPH group tended to be higher (*p* < 0.10). In addition, *Desulfobacteriota* was more abundant in the MCPH group than in the CON group (*p* < 0.05) (Figure 5). At the genus level, the relative abundances of *Barnesiella*, *Clostridia_vadinBB60_group*, and *Parasutterella* in the LCPH group; *Desulfovibrio*, *Lactobacillus*, *Clostridia_vadinBB60_group*, and *Butyricicoccus* in the MCPH group; and *Megamonas* and *Streptococcus* in the HCPH group were higher than those in the CON group (*p* < 0.05) (Figure 6).

In 63-day-old broilers, the abundance of *Synergistota* and *Campylobacterota* was significantly higher in the HCPH group than that in the CON group (*p* < 0.05) (Figure 5). At the genus level, the abundances of *Clostridia_UCG-014* in the LCPH group and *Synergistes* in the HCPH group were higher than those in the CON group (*p* < 0.05). The abundance of *Alistipes* in the LCPH and MCPH groups was lower than that in the CON group (*p* < 0.05) (Figure 6).

To better understand the correlation between crucial gut microbiota alterations and the growth performance (Zhang et al., 2024) of yellow-feather broilers, the Spearman correlation analysis (Supplementary Figure S1) was performed. Spearman correlations showed a varying degrees of correlation between the number of observed species at the genus level and the growth performance. At the genus level, *Lactobacillus* was positively correlated with average daily gain (ADG) in 21-day-old broilers (*p* < 0.05). *Streptococcus* was negatively correlated with ADG and average daily feed intake (ADFI) in 42-day-old broilers (*p* < 0.05).

To better understand the correlation between crucial gut microbiota alterations and serum biochemical indices (Zhang et al., 2024) of yellow-feather broilers, the Spearman correlation analysis (Supplementary Figure S2) was performed. Spearman correlations showed a varying degrees of correlation between the number of observed species at the genus level and serum biochemical indices. At the genus level, *Lactobacillus* was positively correlated with albumin (ALB) and phosphorus (P) and negatively correlated with triglycerides (TG) and total cholesterol (T-CHO) in 21-day-old broilers (*p* < 0.05). *Megamonas* was positively correlated with P and *Butyricicoccus* was positively correlated with ALB and P; *Lactobacillus* and *Butyricicoccus* were negatively correlated with TG; *Clostridia_vadinBB60_group* was negatively correlated with blood urea nitrogen (BUN); *Parasutterella* was negatively correlated with BUN and TG in 42-day-old broilers (*p* < 0.05).

To better understand the correlation between crucial gut microbiota alterations and intestinal morphology (Zhang et al., 2024) of yellow-feather broilers, the Spearman correlation analysis (Supplementary Figures S3–S5) was performed. Spearman correlations showed a varying degrees of correlation between the number of observed species at the genus level and intestinal morphology. *Lactobacillus* was positively correlated with the V/C ratio and negatively correlated with the crypt depth; *Ruminococcus_torques_group* was negatively correlated with the crypt depth of duodenum in 21-day-old broilers (*p* < 0.05). *Lactobacillus* was positively correlated with the villus height of duodenum in 42-day-old broilers (*p* < 0.05). *Phascolarctobacterium* was positively correlated with the villus height of jejunum in 21-day-old broilers (*p* < 0.05). *Megamonas* was positively correlated with the villus height of jejunum in 42-day-old broilers (*p* < 0.05). *Megamonas* was positively correlated with the V/C ratio and negatively correlated with the crypt depth; *Barnesiella* and *Clostridia_vadinBB60_group* were negatively correlated with the V/C ratio of ileum in 42-day-old broilers (*p* < 0.05).

### 3.4 Cecal microbial network analysis

**Figure 7:**
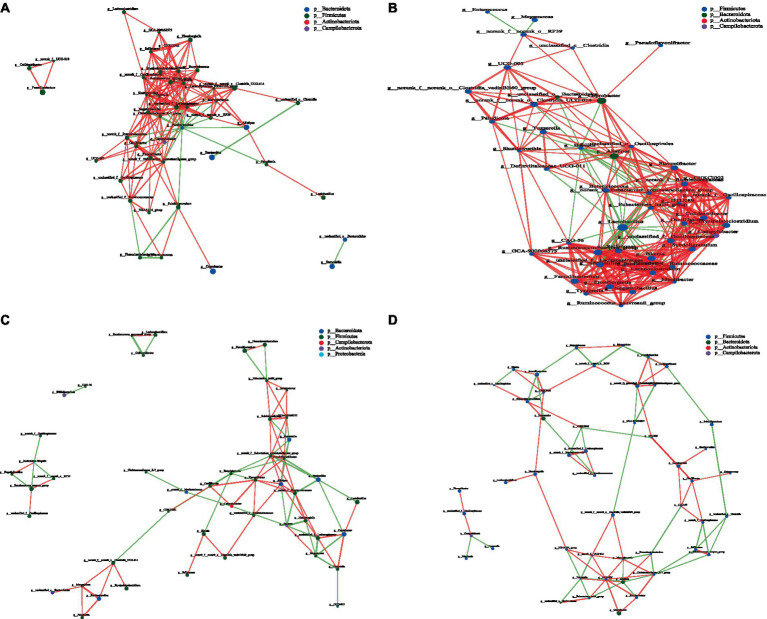
Interaction network diagram of cecal microbiota in 21-day-old yellow-feather broilers. **(A)** The network diagram of the interaction between the cecal microbiota in the CON group. **(B)** The network diagram of the interaction between the cecal microbiota in the LCPH group. **(C)** The network diagram of the interaction between the cecal microbiota in the MCPH group. **(D)** The network diagram of the interaction between the cecal microbiota in the HCPH group.

**Figure 8:**
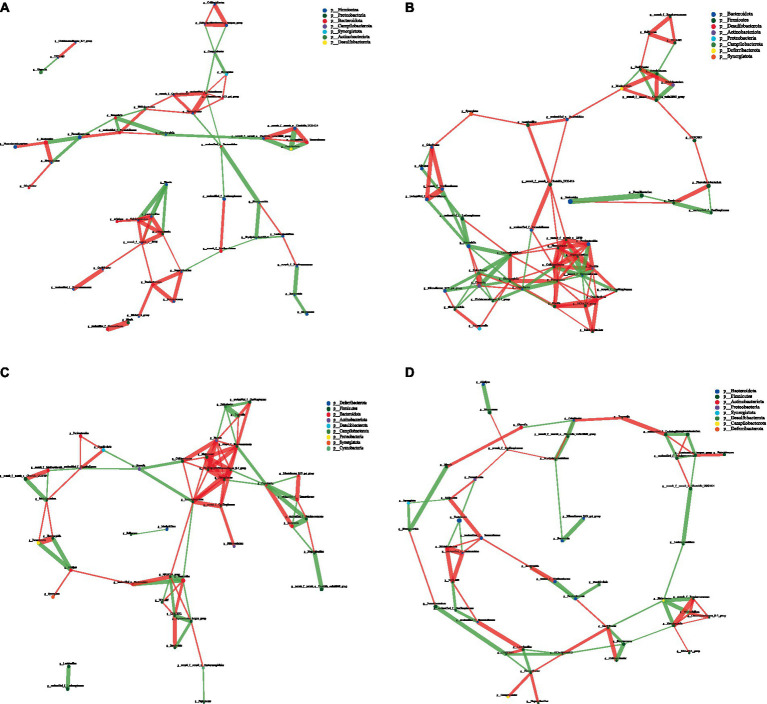
Interaction network diagram of cecal microbiota in 42-day-old yellow-feather broilers. **(A)** The network diagram of the interaction between the cecal microbiota in the CON group. **(B)** The network diagram of the interaction between the cecal microbiota in the LCPH group. **(C)** The network diagram of the interaction between the cecal microbiota in the MCPH group. **(D)** The network diagram of the interaction between the cecal microbiota in the HCPH group.

**Figure 9:**
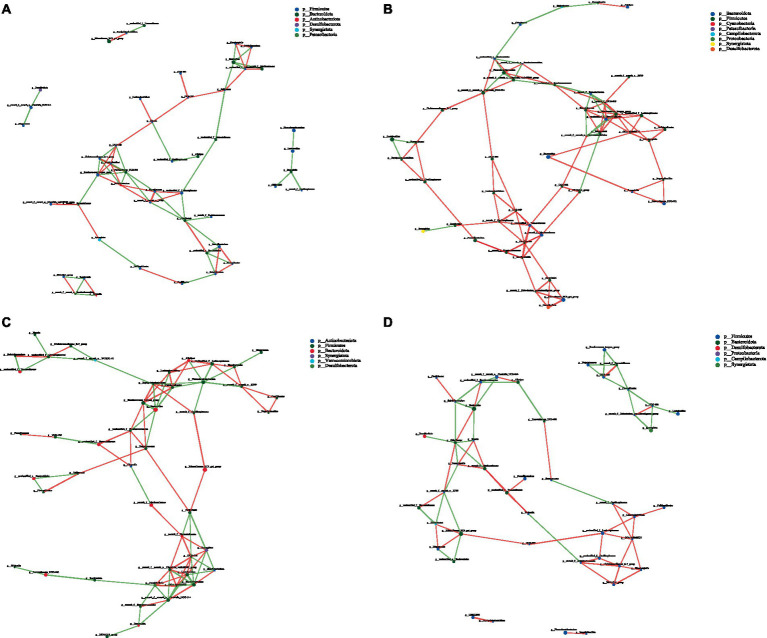
Interaction network diagram of cecal microbiota in 63-day-old yellow-feather broilers. **(A)** The network diagram of the interaction between the cecal microbiota in the CON group. **(B)** The network diagram of the interaction between the cecal microbiota in the LCPH group. **(C)** The network diagram of the interaction between the cecal microbiota in the MCPH group. **(D)** The network diagram of the interaction between the cecal microbiota in the HCPH group.

Network analysis, an effective method for assessing large and complex data, is based on the analysis of species abundance information between different samples; it can reveal the coexisting relationships and interactions between species and highlight the similarities and differences between samples. Therefore, we conducted a network analysis of microorganisms to further study the effects of CPH on cecal microorganisms in yellow-feather broilers. Figures 7–9 and Supplementary Table S5 show the microbial networks in the CON, LCPH, MCPH, and HCPH groups during the three experimental periods.

Among the 21-day-old broilers, the LCPH group had higher proportion of edge_Num and average node connectivity than the CON, MCPH, and HCPH groups. Our results showed that the network complexity in the LCPH group was higher than that in the CON group. In the CON group, the bacterial genera with high number of connected nodes included *Parabacteroides* (degree = 19) and *Tuzzerella*, and *Blautia*, and *Ruminococcus_torques_group* (degree =18). However, the more abundant bacterial genera, such as *Lactobacillus* (degree = 1) and *Coprobacter* (degree = 1), did not exhibit high connectivity. In addition, 165 positive and 24 negative correlations were observed. In the LCPH group, the bacterial genera with the highest number of connected nodes included *norank_f__Eubacterium_coprostanoligenes_group*, *Lactobacillus*, *Lachnoclostridium*, *unclassified_f__Ruminococcaceae*, *Blautia*, *Subdoligranulum*, *unclassified_f__Oscillospiraceae* (degree = 21), *DTU089*, *Sellimonas*, *unclassified_f__Lachnospiraceae*, and *Ruminococcus_torques_group* (degree = 20). Compared to that in the CON group, the node connectivity of *Lactobacillus* (degree = 21) improved. After CPH treatment, 291 positive and 40 negative correlations were observed.

In 42-day-old broilers, the LCPH and MCPH groups had higher proportions of edge_Num and higher average node connectivity than the CON group. In the CON group, the bacterial genera with the highest number of connected nodes included *norank_f__norank_o__RF39*, *unclassified_f__Sutterellaceae* (degree = 6), *Parasutterella*, *Lactobacillus*, *Subdoligranulum*, and *Flavonifractor* (degree = 5). In addition, 31 positive and 29 negative correlations were observed. In the LCPH group, the bacterial genera with the highest number of connected nodes are *Peptococcus* (degree = 12), *Colidextribacter* (degree = 11), *Enorma*, *norank_f__Barnesiellaceae*, *Eisenbergiella* (degree = 10), *norank_f__norank_o__RF39*, and *Flavonifractor* (degree = 9). In addition, 77 positive and 43 negative correlations were observed. In the MCPH group, the bacterial genera with the highest number of connected nodes included *Eisenbergiella* (degree = 6), *Flavonifractor*, *GCA-900066575*, *Ruminococcus_torques_group*, *Helicobacter*, and *Oscillibacter* (degree = 5). Following the CPH treatment, 37 positive and 37 negative correlations were observed.

In 63-day-old broilers, the LCPH and MCPH groups had higher proportions of edge_Num and higher average node connectivity than CON group. In the CON group, the bacterial genera with high number of connected nodes are *Prevotellaceae_UCG-001* (degree = 7), *Ruminococcus_torques_group*, *Bifidobacterium*, and *Odoribacter* (degree = 6). Further, 31 positive and 40 negative correlations were observed. In the LCPH group, the bacterial genera with high number of connected nodes are *norank_f__UCG-010*, *unclassified_f__Barnesiellaceae*, *Ruminococcus_torques_group* (degree = 8), *Butyricicoccus*, *Oscillibacter* (degree = 7), *GCA-900066575*, *Megamonas*, *unclassified_f__Ruminococcaceae*, *norank_f__Muribaculaceae*, *unclassified_f__Tannerellaceae*, *UCG-009*, and *Shuttleworthia* (degree = 6). After CPH treatment, 77 positive and 22 negative correlations were observed. In the MCPH group, the bacterial genera with the highest number of connected nodes included UCG-008, *Faecalibacterium*, *Lactobacillus* (degree = 8), *Phascolarctobacterium*, *Desulfovibrio*, *norank_f__norank_o__Clostridia_UCG-014*, and *norank_f__norank_o__Clostridia_vadinBB60_group* (degree = 7). Moreover, 48 positive and 47 negative correlations were observed.

## 4 Discussion

The highly variable microbiota in poultry gut readily responds to many environmental changes and plays an important role in maintaining normal physiology and promoting general and immune health, which is indicative of the state of the bacteria (Ma et al., 2021; Singh et al., 2022; Stanley et al., 2013, 2014). CPH contains considerable amounts of soluble proteins and small peptides. Our results revealed that the gut microbial structure of the cecum of broilers responded differently to different CPH supplementation levels. Microbiome composition may be influenced by age and gender (Lindenberg et al., 2019). The CPH diet improved the composition of the cecal microflora and increased the microbial diversity of broilers during a specific period; however, the effect of CPH on the cecal microflora gradually decreased with increase in the growth period. Generally, the diversity and richness of the gut microbes vary with the state of the organism; increased diversity contributes to gut microbiome balance and enhances resistance to foreign pathogens. The diversity of microorganisms can be reflected by alpha diversity indices. Higher community richness was reflected by larger Chao1 or ACE values, whereas higher community diversity was indicated by larger Shannon or smaller Simpson values (Xie et al., 2020).

The results of this study showed that CPH significantly affected cecal microbial diversity in yellow-feather broilers; however, this effect was observed only in the early and middle stages of the trial (21-and 42-day-old broilers), indicating that CPH influenced microbial diversity and richness in the early growth stage of the broilers.

Species abundance may be the driving factor for these variations, as the Shannon diversity index remained unaffected; in PCoA with Bray–Curtis distance, the LCPH group of the 21-day-old broilers was distinct from the CON group. However, this was not the case for all the trial stages. The results of the PLS-DA and NMDS analyses further supported that CPH alters the intestinal microbiota of broilers. An apparent clustering of the microbiota was observed in the CPH group during the middle stage.

In our analysis, *Firmicutes* and *Bacteroidetes* were the most abundant phyla in all groups and experimental stages, as previously reported (Oakley et al., 2014; Pandit et al., 2018; Zhang et al., 2022). We generally believe that the *Firmicutes* contains more probiotics, with *Lactobacillus* being one of them. In addition, Firmicutes abundance has been suggested to improve intestinal conditions, particularly when the population of *Proteobacteria* is low (Abudabos et al., 2017; Shirey et al., 2015). The increase and decrease in the relative abundances of *Firmicutes* and *Bacteroidetes*, respectively, may indicate better production performance, which was also verified by our experimental results in the early stages. The abundance of *Verrucomicrobia* is beneficial for inducing regulatory immunity and improving glucose homeostasis; therefore, it is closely related to improvements in metabolism (Cox and Blaser, 2013; Guo et al., 2018; Lindenberg et al., 2019). The increased abundance of *Verrucomicrobia* in 42-day-old broilers improved the composition of cecal microorganisms. No changes were observed at the phylum level in 63-day-old broilers, except for *Synergistota* and *Campylobacterota*. Thus, the effect of CPH on the microphylum levels in broilers was observed mainly during the early and middle stages of the experiment, and some phyla, such as *Firmicutes* and *Verrucomicrobia,* favored broiler metabolism and growth. This also indicates that CPH may alter the metabolism of broilers by altering the intestinal flora, thus changing and improving the growth of broilers.

*Lactobacillus*, an important probiotic that functions to renovate the intestinal immune barrier (Zhang et al., 2022; Zhen et al., 2021), is well known in gastrointestinal health and fermentation communities, and is specifically associated with feed conversion. In addition, it has pathogenic and anti-inflammatory properties due to its ability to produce lactic acid or short-chain fatty acids (SCFAs) (Diepers et al., 2017; Zhao et al., 2019). Our results indicated that CPH exerted a positive effect on Lactobacillus in 21-day-old yellow-feather broilers. The increased abundance in Lactobacillus was beneficial for improving serum biochemical indices, which was beneficial for protein and fat metabolism in broilers. During the test period, ADG and F/G correlated positively and negatively with *Lactobacillus*, respectively. Our results showed that changes in the abundance of *Lactobacillus* improved the cecal microbial composition, and was beneficial for intestinal morphology and growth performance (Zhang et al., 2024) of yellow-feather broilers.

*Phascolarctobacterium* produces SCFAs, including acetate and propionate, and colonizes and exerts beneficial effects on the human gastrointestinal tract (Chen et al., 2021). *Clostridia_vadinBB60_group* may be helpful in reducing the abundance of maleficent bacteria. The increased abundance of *Clostridia_vadinBB60_group* exerts a positive impact on Treg cell counts in mice (Atarashi et al., 2013). *Barnesiella* spp. possesses beneficial antimicrobial properties. SCFA levels may be related to *Barnesiella,* which can produce butyrate and isobutyrate from glucose and positively affect SCFA generation (Gu et al., 2019). Kim et al. (2017) found that the addition of *Clostridia* to the intestinal flora of mouse infants was sufficient to limit colonization by the pathogenic *Enterobacteriaceae*. *Parasutterella* has been linked to multiple health outcomes and is considered a core members of the intestinal flora in humans and mice (Ju et al., 2019). We found that an increase in the abundance of *Clostridia_vadinBB60_group* and *Parasutterella* favored a decrease in BUN in serum. *Desulfovibrio* is a group of sulfate-reducing anaerobic bacteria with more than 30 species and is ubiquitous in nature and animal gastrointestinal tracts (Goldstein et al., 2003). *Butyricicoccus* is beneficial for digestion and nutrient absorption and helps improve metabolic and immune activities (Yang et al., 2021). Our results indicated that the increase of *Butyricicoccus* was conducive to enhancing protein metabolism and P absorption, and may improved fat metabolism of yellow-feather broilers. *Megamonas*, which belongs to the phylum *Firmicutes*, is a unique bacterium present in the cecum microbiota of male chickens; the Na/melibiose symporter and *α*-galactosidase expressed by *Megamonas* are required for breaking down the *α*-1,6 linkage between galactose and glucose in melibiose (Cui et al., 2021; Polansky et al., 2016). The cecal microbiota of male chickens was more dependent on glycan metabolism, as indicated by the high abundance of *Megamonas;* thus, the change in microflora may change the metabolism of broilers, which then affects their growth and development. Our results indicated that increased abundance of *Megamonas* was beneficial for improving intestinal morphology. Therefore, in the early and middle stages of the experiment, an increase in these probiotics was beneficial for improving the cecal microflora structure of yellow-feather broilers, which was conducive for their growth and development. Few non-probiotic changes were also observed. For example, *Streptococcus* comprises ecologically diverse species and includes some pathogenic bacteria (Lee and Andam, 2022), and an increase in its abundance may be detrimental to the organism.

Therefore, CPH increased the relative abundance of some probiotics and affected the cecal microbial diversity and community composition of yellow-feather broilers, which was related to the growth period; this effect gradually decreased with the extension of the growth period. And changes in the abundance of probiotics were beneficial to improve the intestinal morphology and growth performance of yellow-feather broilers. In addition, the CPH supplementation level was an important factor affecting microorganisms abundance; the effects of varying supplementation levels in different periods were not consistent.

Dietary composition may induce different coexisting network topologies in the intestinal microbiota. We found that the effects of CPH on network complexity varied with age. The complexity of the coexistence network varied in the CPH group, which may have been due to changes in the absorption and utilization of nutrients caused by CPH in the intestine, which then changed the interactions between cecal microorganisms. However, the change in network complexity with increasing CPH levels did not follow any trend; for example, on day 21, only the network complexity of the LCPH group was higher than that of the CON group, while that of the MCPH and HCPH groups decreased. However, after day 42, the network complexity of the LCPH and MCPH groups also increased, thus altering the cecal microbial network complexity in response to different levels of CPH, which may also be related to the growth period of broilers. *Lactobacillus* and *Blautia* are beneficial for animal health, and a more complexed correlation with other genera is favorable in broilers. Increased network complexity is helpful in resisting pathogen invasion and external influences (Li et al., 2022; Mendes et al., 2018). Enhancement of the cecal microbial network complexity benefits the health and growth of broilers; the increased network complexity of probiotics may have enhanced this effect.

## 5 Conclusion

This study aimed to determine the effects of CPH on the intestinal microbiota of yellow-feather broilers. We concluded that CPH affects the cecal microbial composition of yellow-feather broilers, increasing the relative abundance of beneficial gut microbiota and enhancing the richness and diversity of the bacterial microbiota in chickens <42 days of age, and changes in the abundance of probiotics were beneficial to improve the intestinal morphology and growth performance. The effect of CPH on intestinal microbiota weakened after 42 days. The LCPH treatment increased the complexity of the cecal microbial network, while the MCPH treatment had the same effect after 42 days, which may have increased resistance to pathogens. The results of this study may act as a reference for the application of CPH to animals.

## Data Availability

The datasets presented in this study can be found in online repositories. The names of the repository/repositories and accession number(s) can be found in the article/Supplementary material.
