# Supplementary material for: Effects of cottonseed meal protein hydrolysate on intestinal microbiota of yellow-feather broilers
Source: Front Microbiol. 2024 Sep 18;15:1434252. doi: 10.3389/fmicb.2024.1434252 (PMC11445190; doi:10.3389/fmicb.2024.1434252)
Supplement: Supplementary file 1 [file Data_Sheet_1.docx]

Supplementary Material

# Supplementary Tables

Supplementary Table 1 Relative abundances (%) of major cecal bacterial compositions at the phylum genus level in yellow-feather broilers treated with different concentrations of CPH

| Phylum | Experimental Treatments (Trt) | | | | SEM | P-value | | | |
| --- | --- | --- | --- | --- | --- | --- | --- | --- | --- |
|  | CON | LCPH | MCPH | HCPH |  | Trt | CON×  LCPH | CON×  MCPH | CON×  HCPH |
| day 21 |  |  |  |  |  |  |  |  |  |
| Firmicutes | 51.08^a^ | 83.33^b^ | 52.42^a^ | 62.88^a^ | 4.168 | 0.012 | 0.003 | 0.892 | 0.236 |
| Bacteroidota | 47.46^a^ | 15.36^b^ | 44.23^a^ | 35.22^ab^ | 4.119 | 0.015 | 0.003 | 0.742 | 0.220 |
| Actinobacteriota | 0.33 | 0.31 | 2.62 | 0.55 | 0.422 | 0.151 | 0.985 | 0.056 | 0.847 |
| Campilobacterota | 0.89 | 0.78 | 0.58 | 0.86 | 0.209 | 0.959 | 0.860 | 0.626 | 0.966 |
| day 42 |  |  |  |  |  |  |  |  |  |
| Firmicutes | 43.14 | 42.12 | 42.21 | 48.64 | 1.920 | 0.608 | 0.856 | 0.868 | 0.336 |
| Bacteroidota | 46.02 | 43.15 | 42.63 | 39.32 | 1.763 | 0.639 | 0.581 | 0.516 | 0.206 |
| Actinobacteriota | 5.42 | 5.86 | 6.05 | 5.60 | 1.034 | 0.997 | 0.889 | 0.843 | 0.955 |
| Desulfobacterota | 1.75 | 2.11 | 2.77 | 2.20 | 0.192 | 0.325 | 0.52 | 0.073 | 0.414 |
| Synergistota | 1.26 | 1.93 | 2.15 | 1.48 | 0.325 | 0.786 | 0.496 | 0.369 | 0.824 |
| Proteobacteria | 1.21 | 1.70 | 1.35 | 1.29 | 0.125 | 0.561 | 0.193 | 0.706 | 0.825 |
| Campilobacterota | 0.48 | 1.93 | 0.59 | 1.05 | 0.411 | 0.614 | 0.239 | 0.932 | 0.637 |
| Deferribacterota | 0.60 | 0.95 | 1.20 | 0.27 | 0.137 | 0.075 | 0.324 | 0.103 | 0.363 |
| Cyanobacteria | 0.04 | 0.17 | 1.00 | 0.07 | 0.220 | 0.373 | 0.825 | 0.133 | 0.954 |
| day 63 |  |  |  |  |  |  |  |  |  |
| Firmicutes | 45.06 | 54.37 | 49.91 | 46.02 | 1.793 | 0.248 | 0.073 | 0.336 | 0.847 |
| Bacteroidota | 47.50 | 37.72 | 42.45 | 44.47 | 1.820 | 0.293 | 0.067 | 0.329 | 0.556 |
| Synergistota | 2.02^a^ | 3.12^ab^ | 2.42^ab^ | 4.20^b^ | 0.329 | 0.087 | 0.210 | 0.637 | 0.018 |
| Desulfobacterota | 2.08 | 2.12 | 1.85 | 2.27 | 0.171 | 0.869 | 0.938 | 0.656 | 0.706 |
| Actinobacteriota | 1.34 | 0.53 | 1.32 | 0.84 | 0.162 | 0.220 | 0.081 | 0.978 | 0.272 |
| Verrucomicrobiota | 0.34 | 0.52 | 0.61 | 0.42 | 0.165 | 0.953 | 0.725 | 0.594 | 0.872 |
| Patescibacteria | 0.59 | 0.27 | 0.52 | 0.15 | 0.097 | 0.352 | 0.256 | 0.813 | 0.124 |
| Spirochaetota | 0.00 | 0.00 | 0.00 | 0.22 | 0.051 | 0.345 | 0.997 | 0.997 | 0.141 |

Experimental Treatments: CON =basal diet, LCPH =diet with 1% CPH, MCPH =diet with 3% CPH, HCPH =diet with 5% CPH;

^a,b,c^ In the same row, values with different letter superscripts mean significant difference (*P*<0.05)

Supplementary Table 2 Relative abundances (%) of major cecal bacterial compositions at the genus level in 21-day-old yellow-feather broilers treated with different concentrations of CPH

| Genus | Experimental Treatments (Trt) | | | | SEM | P-value | | | |
| --- | --- | --- | --- | --- | --- | --- | --- | --- | --- |
|  | CON | LCPH | MCPH | HCPH |  | Trt | CON×  LCPH | CON×  MCPH | CON×  HCPH |
| Lactobacillus | 1.40^a^ | 52.26^b^ | 8.42^a^ | 4.83^a^ | 5.555 | ＜0.001 | ＜0.001 | 0.517 | 0.750 |
| Coprobacter | 17.40 | 9.58 | 15.33 | 21.35 | 2.450 | 0.412 | 0.272 | 0.768 | 0.575 |
| Faecalibacterium | 15.38 | 3.05 | 6.38 | 12.95 | 2.303 | 0.205 | 0.062 | 0.165 | 0.701 |
| Bacteroides | 15.25 | 0.12 | 14.42 | 7.74 | 4.488 | 0.635 | 0.260 | 0.950 | 0.572 |
| Alistipes | 8.43 | 5.53 | 6.14 | 5.95 | 1.089 | 0.806 | 0.379 | 0.486 | 0.451 |
| Ruminococcus_torques_group | 2.52 | 4.25 | 4.99 | 2.80 | 0.627 | 0.476 | 0.344 | 0.183 | 0.876 |
| unclassified_f__Lachnospiraceae | 2.48 | 2.12 | 3.64 | 3.02 | 0.325 | 0.399 | 0.703 | 0.222 | 0.561 |
| norank_f__norank_o__Clostridia_UCG-014 | 4.46 | 1.48 | 1.50 | 3.80 | 0.830 | 0.482 | 0.224 | 0.226 | 0.782 |
| norank_o__Clostridia_vadinBB60_group | 3.36^ac^ | 0.46^b^ | 1.08^ab^ | 3.86^c^ | 0.471 | 0.013 | 0.015 | 0.050 | 0.654 |
| Barnesiella | 4.79 | 0.00 | 2.97 | 0.01 | 1.091 | 0.338 | 0.132 | 0.557 | 0.133 |
| Ruminococcaceae | 1.50 | 0.96 | 1.88 | 2.76 | 0.277 | 0.125 | 0.463 | 0.614 | 0.101 |
| Eisenbergiella | 1.64 | 1.53 | 1.54 | 2.32 | 0.353 | 0.857 | 0.922 | 0.930 | 0.526 |
| Erysipelatoclostridium | 1.28 | 1.25 | 2.56 | 1.85 | 0.417 | 0.678 | 0.979 | 0.305 | 0.645 |
| Subdoligranulum | 1.34 | 1.02 | 1.65 | 2.79 | 0.579 | 0.746 | 0.854 | 0.854 | 0.402 |
| Blautia | 1.13 | 2.19 | 2.30 | 0.84 | 0.356 | 0.379 | 0.307 | 0.257 | 0.771 |
| unclassified_f__Oscillospiraceae | 0.75 | 1.38 | 1.34 | 2.26 | 0.269 | 0.269 | 0.404 | 0.434 | 0.055 |
| Flavonifractor | 0.91 | 1.30 | 0.76 | 1.62 | 0.232 | 0.579 | 0.562 | 0.824 | 0.304 |
| Colidextribacter | 1.07 | 1.00 | 0.42 | 2.00 | 0.223 | 0.082 | 0.909 | 0.274 | 0.121 |
| Phascolarctobacterium | 0.49^a^ | 0.00^a^ | 3.55^b^ | 0.21^a^ | 0.432 | 0.003 | 0.613 | 0.004 | 0.773 |
| Butyricicoccus | 1.06 | 0.74 | 0.61 | 1.70 | 0.206 | 0.256 | 0.580 | 0.437 | 0.276 |

Experimental Treatments: CON =basal diet, LCPH =diet with 1% CPH, MCPH =diet with 3% CPH, HCPH =diet with 5% CPH;

^a,b,c^ In the same row, values with different letter superscripts mean significant difference (P<0.05)

Supplementary Table 3 Relative abundances (%) of major cecal bacterial compositions at the genus level in 42-day-old yellow-feather broilers treated with different concentrations of CPH

| Genus | Experimental Treatments (Trt) | | | | SEM | P-value | | | |
| --- | --- | --- | --- | --- | --- | --- | --- | --- | --- |
|  | CON | LCPH | MCPH | HCPH |  | Trt | CON×  LCPH | CON×  MCPH | CON×  HCPH |
| Bacteroides | 33.79 | 22.70 | 27.99 | 26.98 | 1.996 | 0.279 | 0.058 | 0.305 | 0.231 |
| Faecalibacterium | 16.57 | 4.97 | 7.79 | 4.85 | 2.193 | 0.191 | 0.064 | 0.154 | 0.062 |
| Phascolarctobacterium | 4.86 | 7.09 | 7.49 | 7.57 | 0.675 | 0.467 | 0.261 | 0.187 | 0.175 |
| Olsenella | 3.96 | 3.81 | 3.50 | 4.74 | 0.988 | 0.979 | 0.962 | 0.880 | 0.797 |
| unclassified_f__Lachnospiraceae | 3.50 | 3.63 | 3.01 | 4.32 | 0.275 | 0.427 | 0.867 | 0.536 | 0.303 |
| Barnesiella | 1.84^a^ | 7.82^b^ | 2.73^a^ | 1.42^a^ | 0.759 | 0.003 | 0.002 | 0.594 | 0.798 |
| norank_f__norank_o__Clostridia_UCG-014 | 3.33 | 3.82 | 2.87 | 3.43 | 0.360 | 0.848 | 0.652 | 0.671 | 0.928 |
| norank_f__Muribaculaceae | 2.33 | 1.77 | 2.52 | 2.66 | 0.347 | 0.831 | 0.591 | 0.853 | 0.750 |
| unclassified_f__Tannerellaceae | 2.10 | 1.93 | 2.15 | 2.91 | 0.390 | 0.839 | 0.883 | 0.968 | 0.495 |
| Megamonas | 1.27^a^ | 0.67^a^ | 2.08^a^ | 4.96^b^ | 0.543 | 0.015 | 0.640 | 0.535 | 0.009 |
| Desulfovibrio | 1.67 | 2.03 | 2.71 | 2.16 | 0.193 | 0.307 | 0.510 | 0.068 | 0.374 |
| Parabacteroides | 1.71 | 3.03 | 2.18 | 1.61 | 0.333 | 0.442 | 0.179 | 0.626 | 0.914 |
| Ruminococcus_torques_group | 1.38 | 2.05 | 1.61 | 2.24 | 0.205 | 0.453 | 0.266 | 0.692 | 0.155 |
| Synergistes | 1.26 | 1.93 | 2.15 | 1.48 | 0.325 | 0.786 | 0.496 | 0.369 | 0.824 |
| Lactobacillus | 0.89 | 2.13 | 1.98 | 1.62 | 0.264 | 0.373 | 0.112 | 0.160 | 0.342 |
| norank_o__Clostridia_vadinBB60_group | 0.92^a^ | 2.29^b^ | 1.76^b^ | 1.20^a^ | 0.141 | 0.000 | 0.000 | 0.005 | 0.322 |
| Blautia | 1.01 | 2.06 | 1.63 | 0.99 | 0.222 | 0.259 | 0.099 | 0.318 | 0.986 |
| unclassified_f__Barnesiellaceae | 1.58 | 1.26 | 1.67 | 0.47 | 0.317 | 0.555 | 0.735 | 0.916 | 0.240 |
| Butyricicoccus | 0.86 | 1.26 | 1.69 | 0.95 | 0.149 | 0.197 | 0.334 | 0.055 | 0.833 |
| Alistipes | 1.06 | 1.30 | 1.21 | 1.18 | 0.084 | 0.811 | 0.344 | 0.552 | 0.631 |
| Streptococcus | 0.42^a^ | 0.05^a^ | 0.02^a^ | 3.99^b^ | 0.493 | 0.003 | 0.732 | 0.711 | 0.003 |
| Parasutterella | 0.85 | 1.39 | 1.14 | 0.93 | 0.099 | 0.229 | 0.061 | 0.310 | 0.767 |

Experimental Treatments: CON =basal diet, LCPH =diet with 1% CPH, MCPH =diet with 3% CPH, HCPH =diet with 5% CPH;

^a,b,c^ In the same row, values with different letter superscripts mean significant difference (P<0.05)

Supplementary Table 4 Relative abundances (%) of major cecal acterial compositions at the genus level in 63-day-old yellow-feather broilers treated with different concentrations of CPH

| Genus | Experimental Treatments (Trt) | | | | SEM | P-value | | | |
| --- | --- | --- | --- | --- | --- | --- | --- | --- | --- |
|  | CON | LCPH | MCPH | HCPH |  | Trt | CON×  LCPH | CON×  MCPH | CON×  HCPH |
| Bacteroides | 19.86 | 12.99 | 15.21 | 17.23 | 1.565 | 0.479 | 0.140 | 0.310 | 0.562 |
| Rikenellaceae_RC9_gut_group | 11.41 | 11.07 | 10.98 | 12.12 | 0.941 | 0.977 | 0.906 | 0.880 | 0.805 |
| Phascolarctobacterium | 6.58 | 4.87 | 5.87 | 7.58 | 0.822 | 0.720 | 0.486 | 0.771 | 0.683 |
| Lactobacillus | 5.20 | 10.43 | 3.18 | 4.32 | 2.006 | 0.619 | 0.380 | 0.732 | 0.882 |
| Ruminococcus_torques_group | 3.36 | 4.24 | 5.39 | 2.88 | 0.480 | 0.277 | 0.515 | 0.141 | 0.721 |
| norank_f__norank_o__Clostridia_UCG-014 | 3.19^a^ | 5.21^b^ | 3.27^a^ | 2.79^a^ | 0.310 | 0.015 | 0.011 | 0.908 | 0.591 |
| unclassified_f__Lachnospiraceae | 3.16 | 3.07 | 3.89 | 3.16 | 0.245 | 0.637 | 0.900 | 0.317 | 0.992 |
| Faecalibacterium | 3.11 | 3.56 | 4.54 | 1.96 | 0.693 | 0.648 | 0.826 | 0.489 | 0.575 |
| Synergistes | 2.02^a^ | 3.12^ab^ | 2.42^ab^ | 4.20^b^ | 0.329 | 0.087 | 0.210 | 0.637 | 0.018 |
| norank_f__Muribaculaceae | 3.26 | 3.01 | 2.61 | 2.80 | 0.266 | 0.859 | 0.752 | 0.418 | 0.569 |
| unclassified_f__Barnesiellaceae | 1.62 | 2.59 | 3.52 | 2.46 | 0.535 | 0.689 | 0.541 | 0.238 | 0.594 |
| unclassified_f__Tannerellaceae | 1.47 | 1.81 | 1.71 | 2.81 | 0.248 | 0.247 | 0.620 | 0.722 | 0.064 |
| Desulfovibrio | 1.95 | 1.96 | 1.74 | 2.15 | 0.168 | 0.879 | 0.982 | 0.686 | 0.689 |
| Prevotellaceae_UCG-001 | 2.08 | 1.83 | 2.53 | 0.65 | 0.406 | 0.427 | 0.830 | 0.706 | 0.227 |
| Alistipes | 1.99 | 1.34 | 1.37 | 2.05 | 0.126 | 0.060 | 0.053 | 0.067 | 0.867 |
| norank_o__Clostridia_vadinBB60_group | 1.31 | 1.94 | 1.48 | 2.02 | 0.145 | 0.233 | 0.122 | 0.656 | 0.086 |
| unclassified_o__Bacteroidales | 2.25^a^ | 0.63^b^ | 1.74^ab^ | 1.47^ab^ | 0.255 | 0.146 | 0.027 | 0.464 | 0.265 |
| CHKCI001 | 0.74^a^ | 1.46^ab^ | 1.36^ab^ | 1.86^b^ | 0.152 | 0.059 | 0.074 | 0.121 | 0.009 |
| unclassified_f__Oscillospiraceae | 0.87 | 1.62 | 1.05 | 1.48 | 0.144 | 0.216 | 0.070 | 0.640 | 0.134 |
| Odoribacter | 1.49 | 0.79 | 1.12 | 1.46 | 0.283 | 0.817 | 0.413 | 0.658 | 0.973 |
| Megamonas | 0.95 | 1.14 | 1.26 | 1.28 | 0.271 | 0.977 | 0.822 | 0.712 | 0.697 |
| Butyricicoccus | 0.80 | 1.17 | 1.19 | 1.37 | 0.142 | 0.568 | 0.379 | 0.353 | 0.176 |
| Colidextribacter | 1.11 | 1.16 | 1.19 | 0.96 | 0.078 | 0.766 | 0.834 | 0.737 | 0.526 |
| UCG-005 | 0.73 | 1.21 | 0.98 | 1.16 | 0.107 | 0.396 | 0.126 | 0.422 | 0.171 |

Experimental Treatments: CON =basal diet, LCPH =diet with 1% CPH, MCPH =diet with 3% CPH, HCPH =diet with 5% CPH;

^a,b,c^ In the same row, values with different letter superscripts mean significant difference (P<0.05)

Supplementary Table 5 The topological properties of the network are obtained from different treatment groups

| Topological properties | CON | LCPH | MCPH | HCPH |
| --- | --- | --- | --- | --- |
| 21 |  |  |  |  |
| Node_Num^1^ | 45 | 47 | 48 | 49 |
| Edge_Num^2^ | 189 | 331 | 88 | 89 |
| Average node connectivity^3^ | 8.400 | 14.085 | 3.667 | 3.633 |
| 42 |  |  |  |  |
| Node_Num^1^ | 46 | 47 | 46 | 49 |
| Edge_Num^2^ | 70 | 120 | 88 | 74 |
| Average node connectivity^3^ | 3.043 | 5.106 | 3.826 | 3.020 |
| 63 |  |  |  |  |
| Node_Num^1^ | 48 | 49 | 48 | 46 |
| Edge_Num^2^ | 71 | 99 | 95 | 70 |
| Average node connectivity^3^ | 2.958 | 4.041 | 3.958 | 3.043 |
| 1.Number of OTUs with at least one correlation ≥ 0.5 or ≤ -0.5 and that are statistically significant (P< 0.05). | | | | |
| 2.Number of strong and significant correlations between nodes. | | | | |
| 3.Node connectivity showing how many connections (on average) each node has to another unique node in the network. | | | | |

# Supplementary Figures


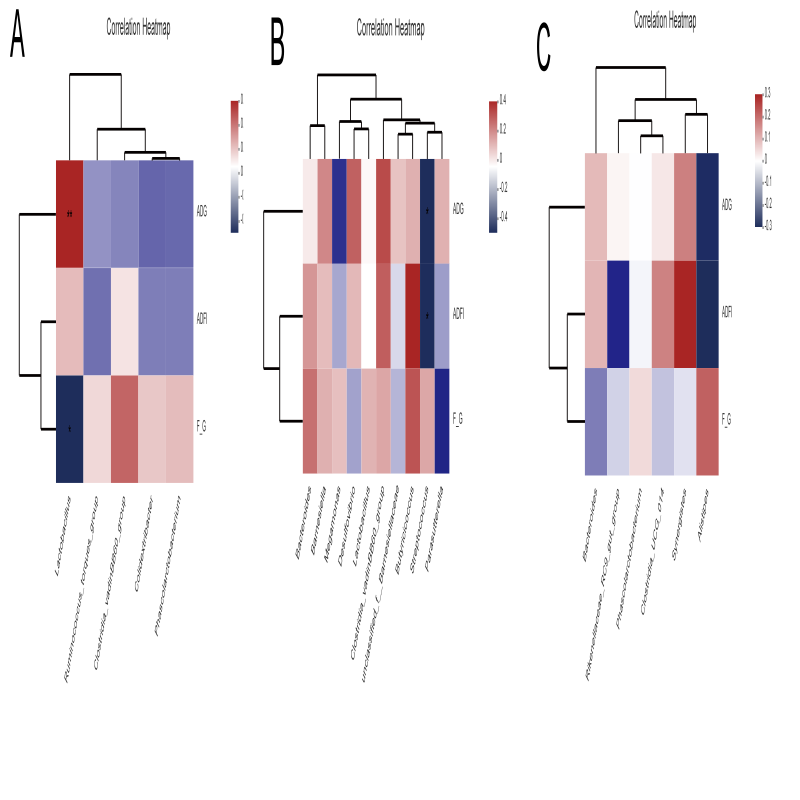


**Supplementary** **Figure 1** Correlation between crucial gut microbiota and growth performance (Zhang et al. 2024). (A) Correlation between crucial gut microbiota and growth performance in 21-day-old yellow-feather broilers. (B) Correlation between crucial gut microbiota and growth performance in 42-day-old yellow-feather broiler. (C) Correlation between crucial gut microbiota and growth performance in 63-day-old yellow-feather broiler. Line color represents the direction of correlation: red (positive), blue (negative), the deeper the color, and the stronger the correlation. The asterisk (*) level presented the degree of significant difference, *P<0.05, **P<0.01 and ***P<0.001. Each mean represents six samples. CON =basal diet, LCPH =diet with 1% CPH, MCPH =diet with 3% CPH, HCPH =diet with 5% CPH.


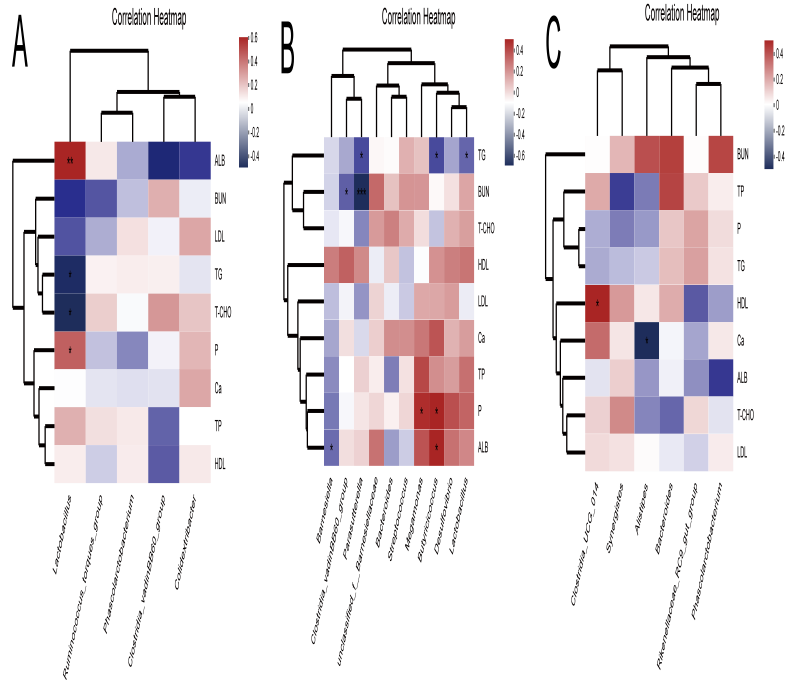


**Supplementary Figure 2** Correlation between crucial gut microbiota and serum biochemical indices (Zhang et al. 2024). (A) Correlation between crucial gut microbiota and serum biochemical indices in 21-day-old yellow-feather broilers. (B) Correlation between crucial gut microbiota and serum biochemical indices in 42-day-old yellow-feather broiler. (C) Correlation between crucial gut microbiota and serum biochemical indices in 63-day-old yellow-feather broiler. Line color represents the direction of correlation: red (positive), blue (negative), the deeper the color, and the stronger the correlation. The asterisk (*) level presented the degree of significant difference, *P<0.05, **P<0.01 and ***P<0.001. Each mean represents six samples. CON =basal diet, LCPH =diet with 1% CPH, MCPH =diet with 3% CPH, HCPH =diet with 5% CPH.


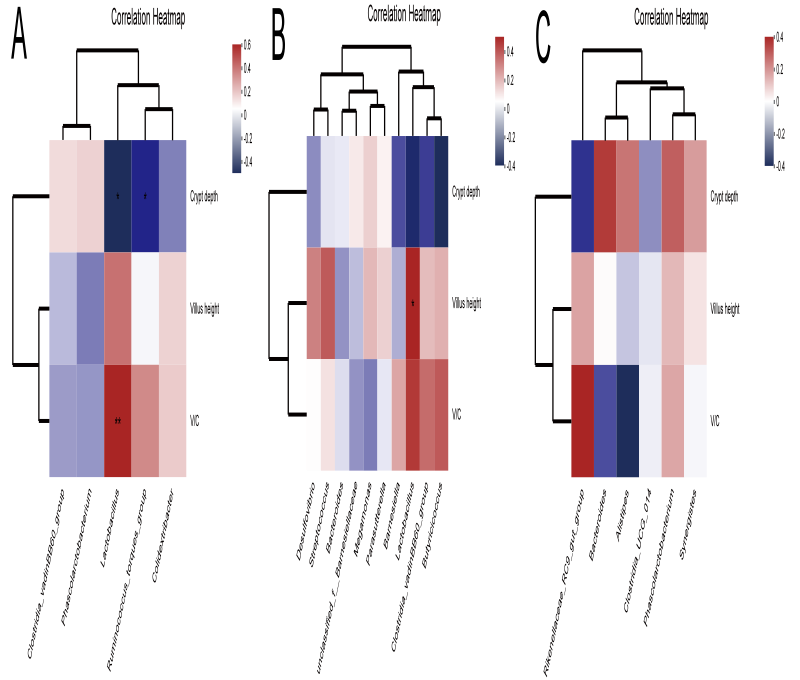


**Supplementary Figure 3** Correlation between crucial gut microbiota and duodenum morphology (Zhang et al. 2024). (A) Correlation between crucial gut microbiota and duodenum morphology in 21-day-old yellow-feather broilers. (B) Correlation between crucial gut microbiota and duodenum morphology in 42-day-old yellow-feather broiler. (C) Correlation between crucial gut microbiota and duodenum morphology in 63-day-old yellow-feather broiler. Line color represents the direction of correlation: red (positive), blue (negative), the deeper the color, and the stronger the correlation. The asterisk (*) level presented the degree of significant difference, *P<0.05, **P<0.01 and ***P<0.001. Each mean represents six samples. CON =basal diet, LCPH =diet with 1% CPH, MCPH =diet with 3% CPH, HCPH =diet with 5% CPH.


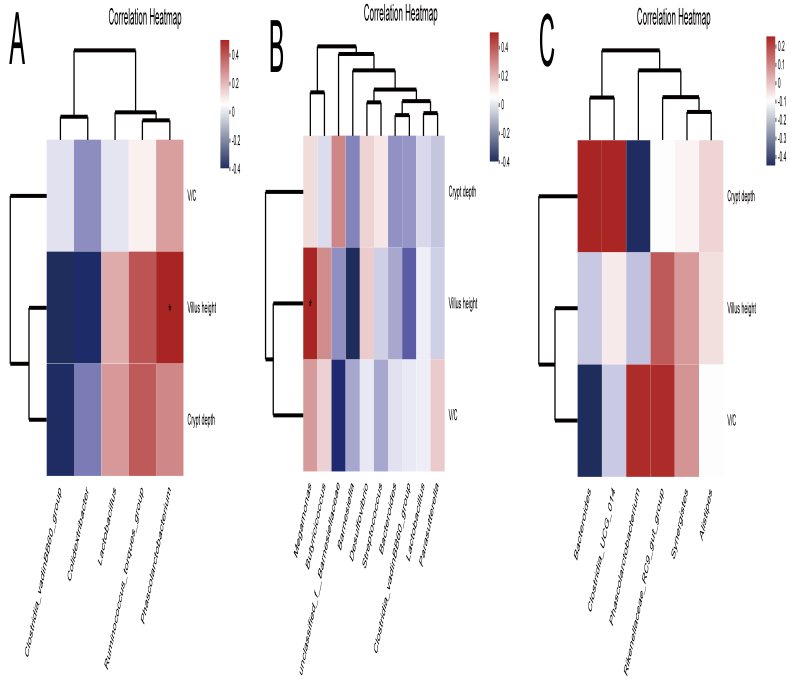
**Supplementary Figure 4** Correlation between crucial gut microbiota and jejunum morphology (Zhang et al. 2024). (A) Correlation between crucial gut microbiota and jejunum morphology in 21-day-old yellow-feather broilers. (B) Correlation between crucial gut microbiota and jejunum morphology in 42-day-old yellow-feather broiler. (C) Correlation between crucial gut microbiota and jejunum morphology in 63-day-old yellow-feather broiler. Line color represents the direction of correlation: red (positive), blue (negative), the deeper the color, and the stronger the correlation. The asterisk (*) level presented the degree of significant difference, *P<0.05, **P<0.01 and ***P<0.001. Each mean represents six samples. CON =basal diet, LCPH =diet with 1% CPH, MCPH =diet with 3% CPH, HCPH =diet with 5% CPH.


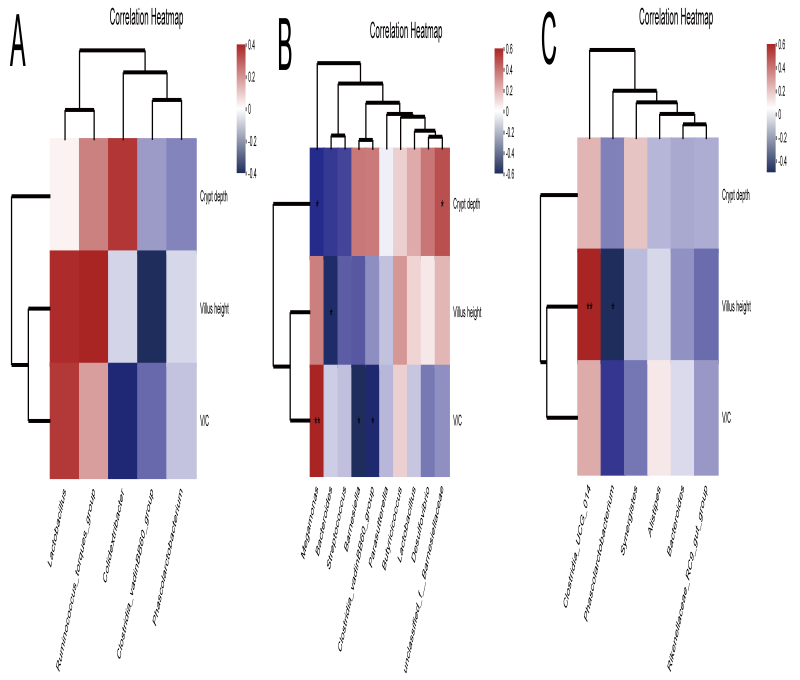


**Supplementary Figure 5** Correlation between crucial gut microbiota and ileum morphology (Zhang et al. 2024). (A) Correlation between crucial gut microbiota and ileum morphology in 21-day-old yellow-feather broilers. (B) Correlation between crucial gut microbiota and ileum morphology in 42-day-old yellow-feather broiler. (C) Correlation between crucial gut microbiota and ileum morphology in 63-day-old yellow-feather broiler. Line color represents the direction of correlation: red (positive), blue (negative), the deeper the color, and the stronger the correlation. The asterisk (*) level presented the degree of significant difference, *P<0.05, **P<0.01 and ***P<0.001. Each mean represents six samples. CON =basal diet, LCPH =diet with 1% CPH, MCPH =diet with 3% CPH, HCPH =diet with 5% CPH.


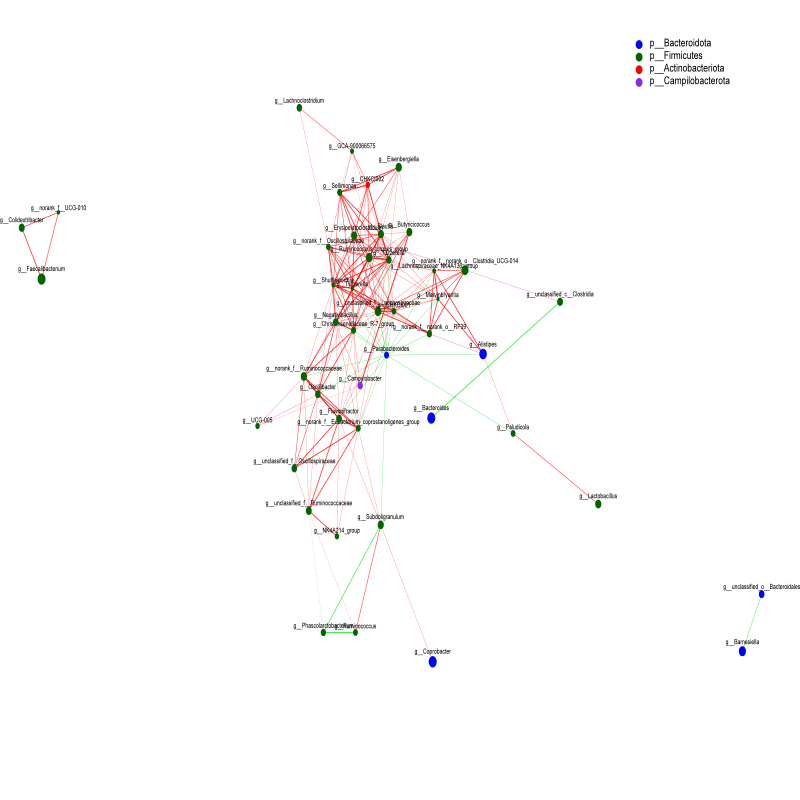


**Supplementary Figure 6** Interaction network diagram of cecal microbiota in the CON group in 21-day-old yellow-feather broilers.


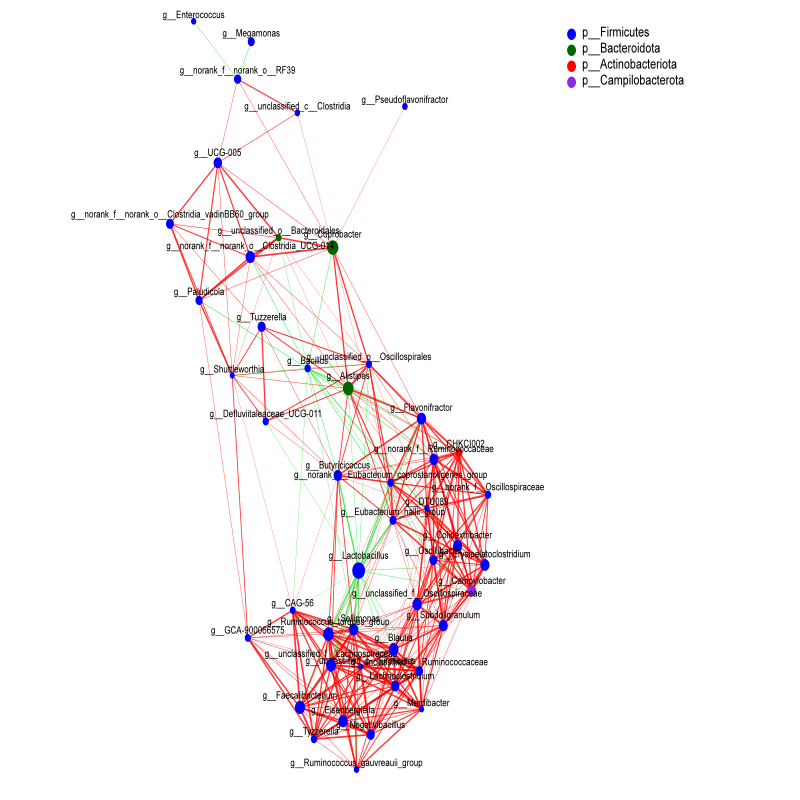


**Supplementary Figure 7** Interaction network diagram of cecal microbiota in the LCPH group in 21-day-old yellow-feather broilers.

**
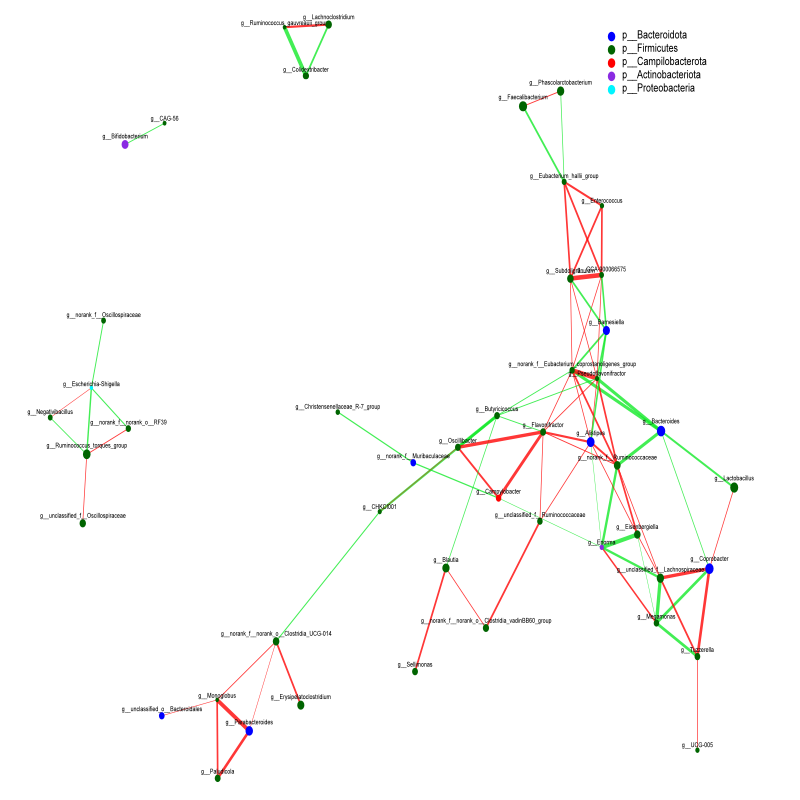
**

**Supplementary Figure 8** Interaction network diagram of cecal microbiota in the MCPH group in 21-day-old yellow-feather broilers.


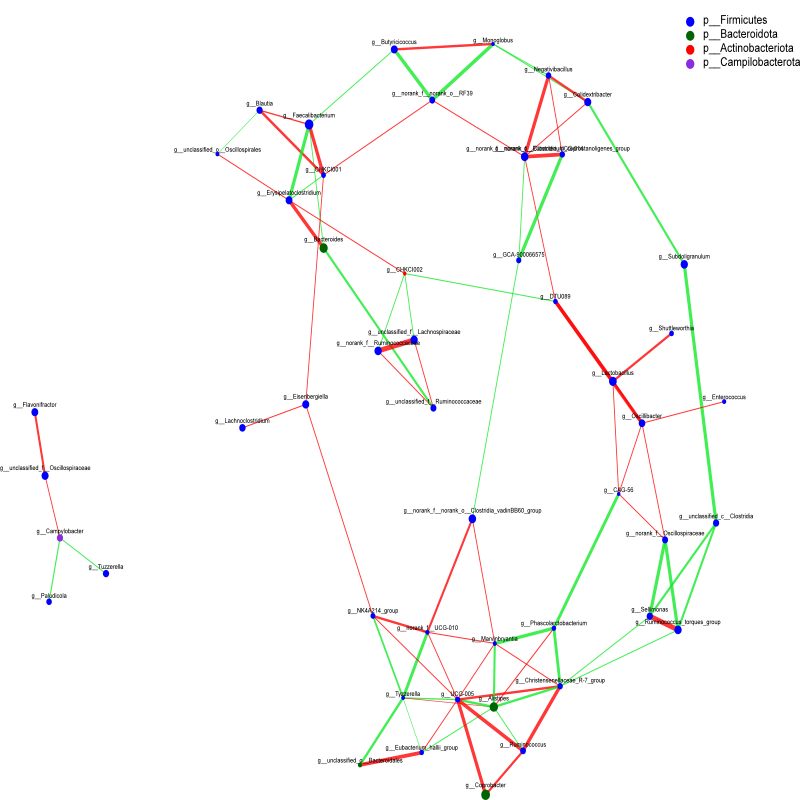


**Supplementary Figure 9** Interaction network diagram of cecal microbiota in the HCPH group in 21-day-old yellow-feather broilers.


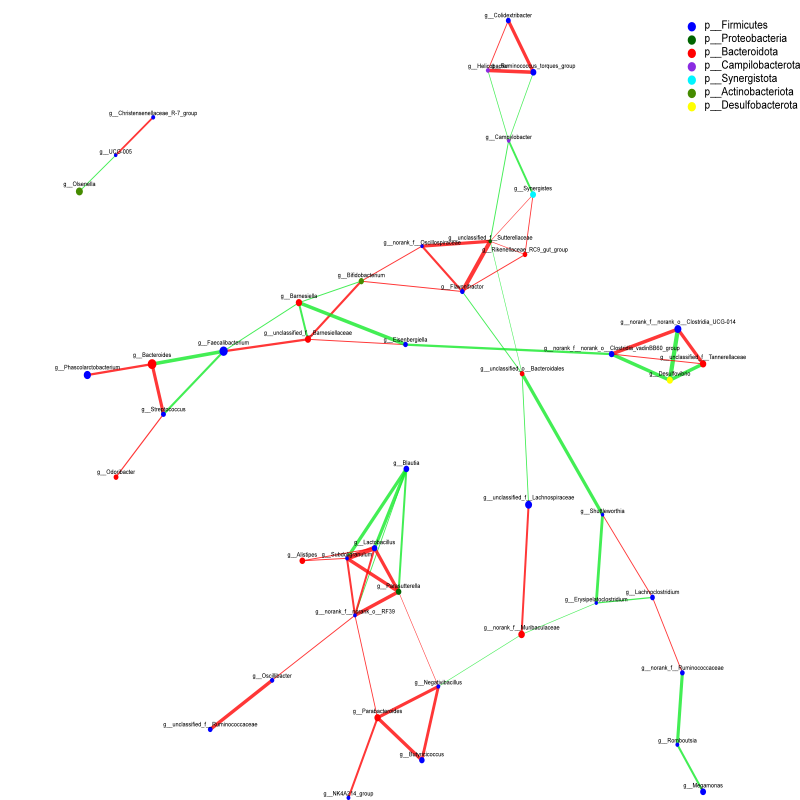


**Supplementary Figure 10** Interaction network diagram of cecal microbiota in the CON group in 42-day-old yellow-feather broilers.


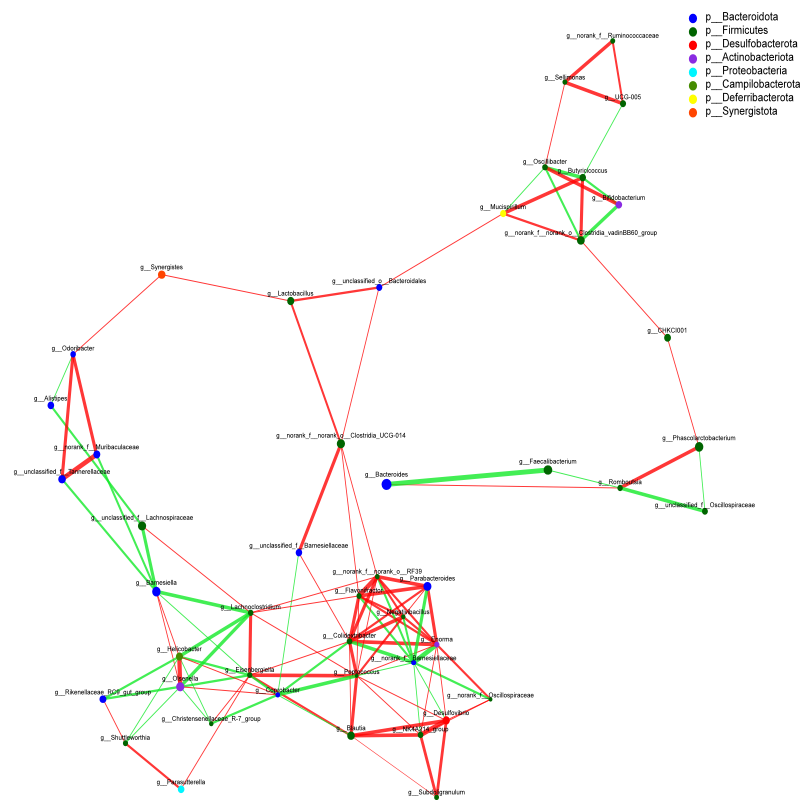


**Supplementary Figure 11** Interaction network diagram of cecal microbiota in the LCPH group in 42-day-old yellow-feather broilers.


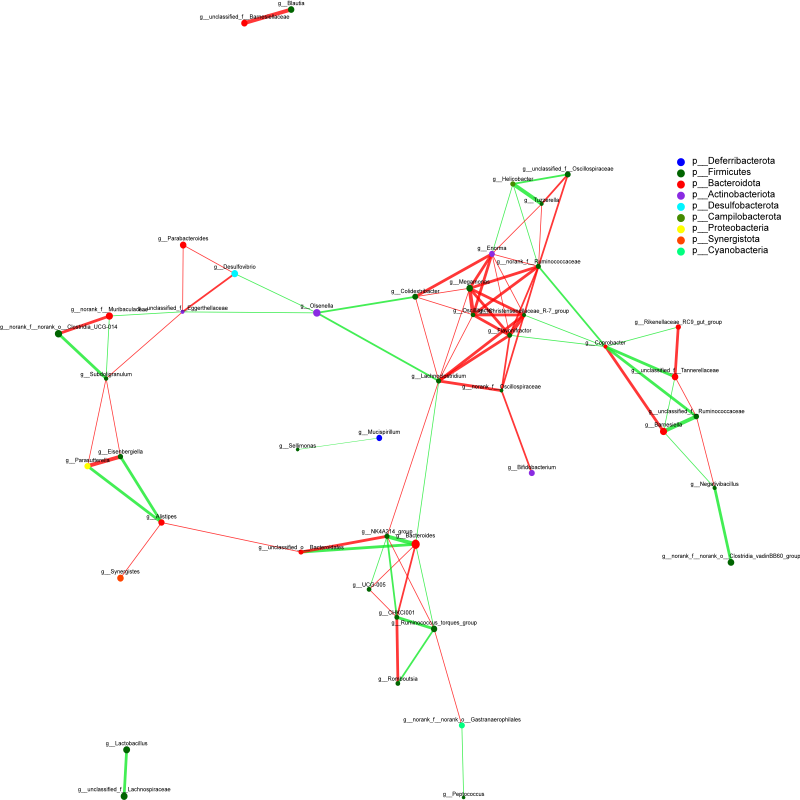


**Supplementary Figure 12** Interaction network diagram of cecal microbiota in the MCPH group in 42-day-old yellow-feather broilers.


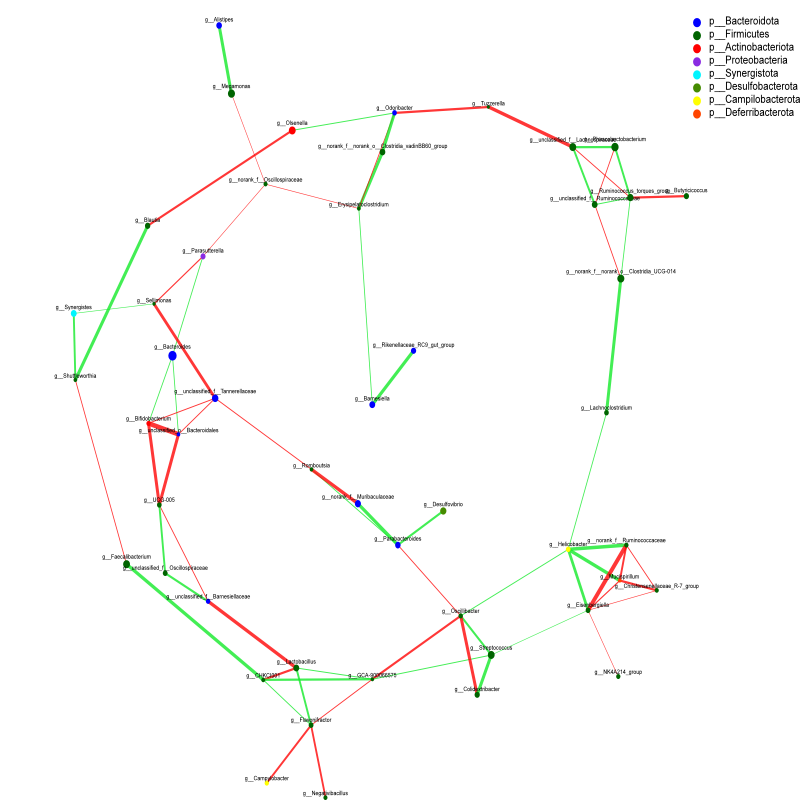


**Supplementary Figure 13** Interaction network diagram of cecal microbiota in the HCPH group in 42-day-old yellow-feather broilers.


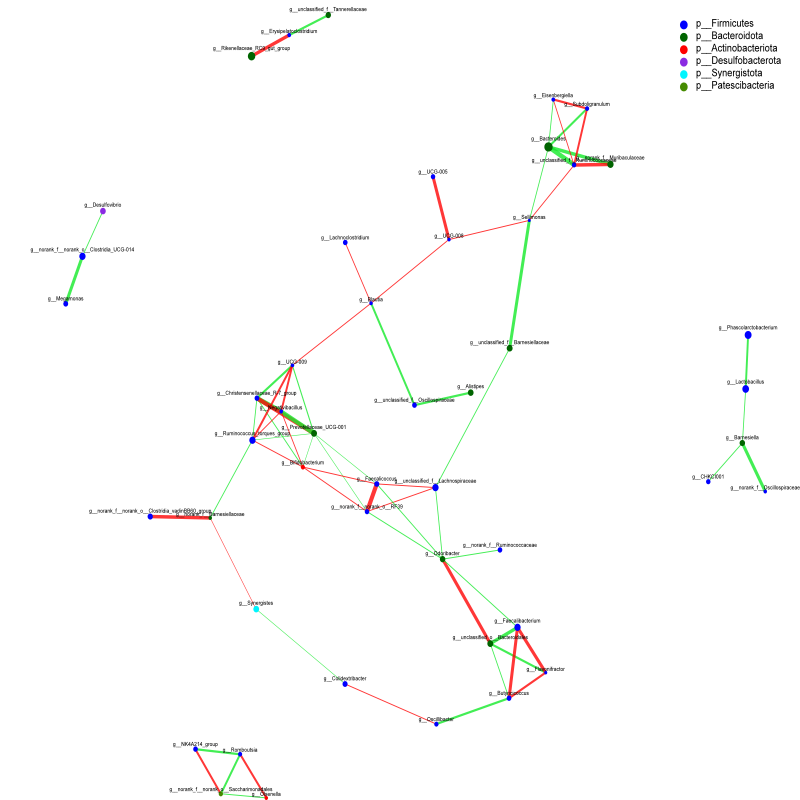


**Supplementary Figure 14** Interaction network diagram of cecal microbiota in the CON group in 63-day-old yellow-feather broilers.


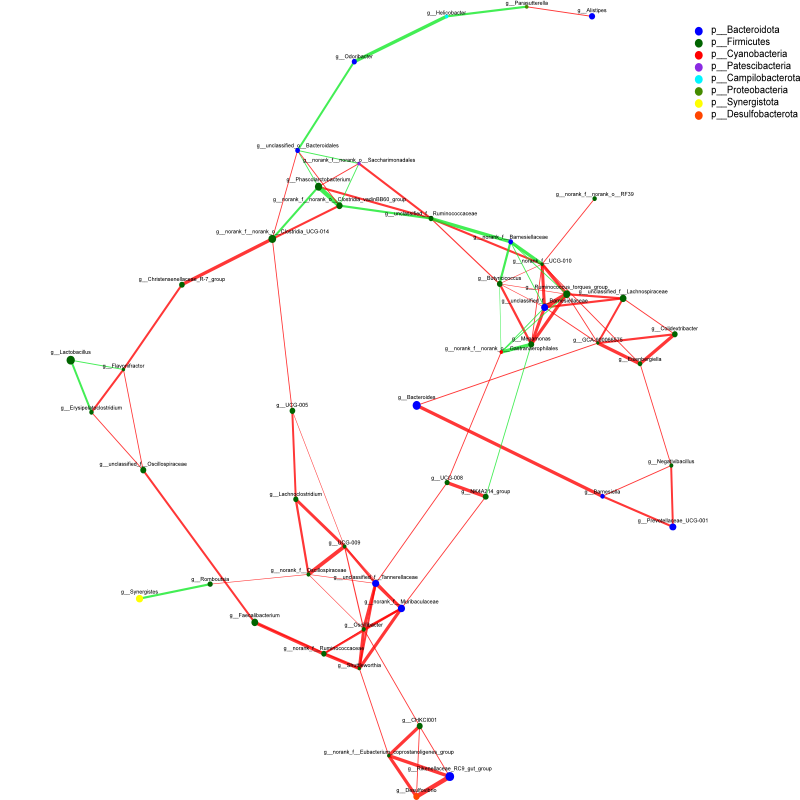


**Supplementary Figure 15** Interaction network diagram of cecal microbiota in the LCPH group in 63-day-old yellow-feather broilers.


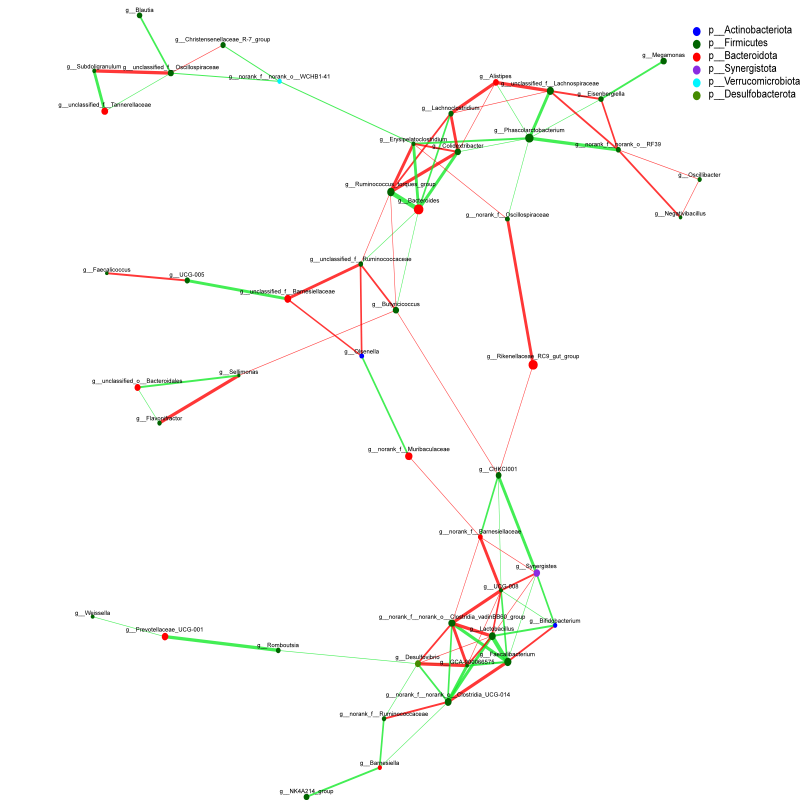


**Supplementary Figure 16** Interaction network diagram of cecal microbiota in the MCPH group in 63-day-old yellow-feather broilers.


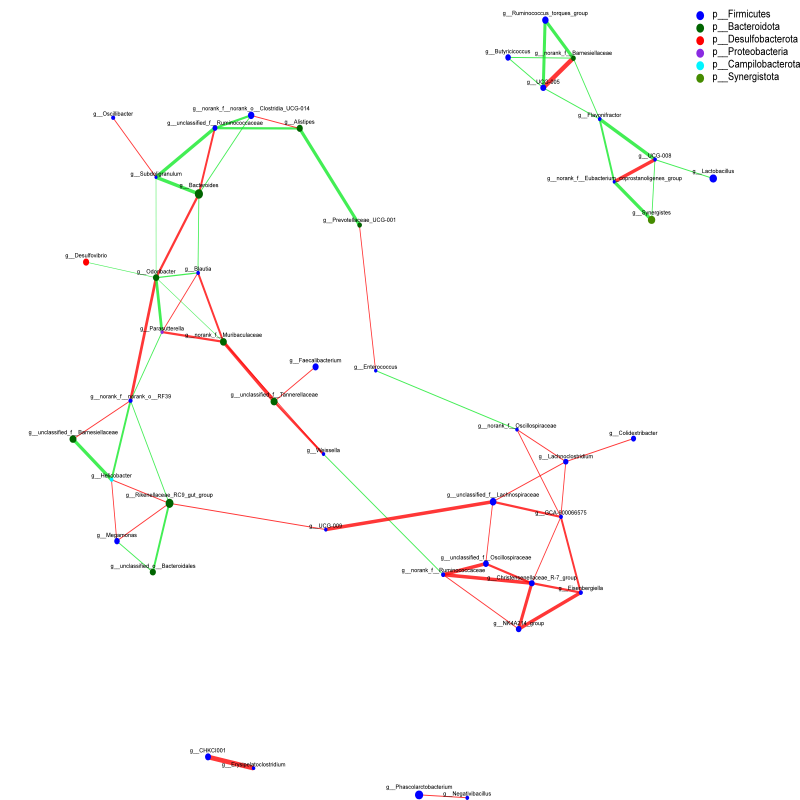


**Supplementary Figure 17** Interaction network diagram of cecal microbiota in the HCPH group in 63-day-old yellow-feather broilers.
